# Inhaling Eugenol Inhibits NAFLD by Activating the Hepatic Ectopic Olfactory Receptor Olfr544 and Modulating the Gut Microbiota

**DOI:** 10.1002/advs.202510321

**Published:** 2025-08-20

**Authors:** Xiao‐Ran Wang, Zhan‐Zhan Li, Shu‐Ding Sun, Ya‐Gang Song, Jin‐Xin Miao, Xiang‐Xiang Wu, Yong‐Li Han, Xiao‐Lei Zhang, Wen‐Jing Chen, Qing‐Hua Wang, Yu Zhang, Yiping Fu, Yu‐ting Liu, Lin‐yan Lang, Wen‐Xia Zhao, Ming‐San Miao

**Affiliations:** ^1^ First Clinical Medical College Henan University of Chinese Medicine Zhengzhou 450046 China; ^2^ Department of Digestive Diseases The First Affiliated Hospital of Henan University of Chinese Medicine Zhengzhou 450046 China; ^3^ Academy of Chinese Medicine Sciences Henan University of Chinese Medicine Zhengzhou 450046 China; ^4^ Henan Collaborative Innovation Center for Research and Development on the Whole Industry Chain of Yu‐Yao Zhengzhou 450046 China; ^5^ Acupuncture Department The First Affiliated Hospital of Henan University of Chinese Medicine Zhengzhou 450046 China; ^6^ Pharmacy College Henan University of Chinese Medicine Zhengzhou 450046 China

**Keywords:** eugenol, gut microbiota, NAFLD, olfactory receptor, volatile oils

## Abstract

Non‐alcoholic fatty liver disease (NAFLD) is a major public health threat with currently limited therapeutic options. Inhalation therapy shows promise for treating metabolic disorders due to rapid absorption and high patient adherence, though relevant medications remain scarce. Eugenol (EUG), the primary component of Syzygium aromaticum volatile oil, emerges as a promising NAFLD inhibitor from lipid‐lowering aromatic Chinese medicine screening. EUG elicited significant anti‐steatotic effects in both cultured hepatocytes and comprehensive high‐fat diet‐induced NAFLD animal models. Mechanistically, EUG targeted the activation of the hepatic ectopic olfactory receptor Olfr544 and up‐regulated its downstream cyclic adenosine monophosphate (cAMP)/protein kinase A (PKA)/cAMP response element binding protein signaling pathway, which further promoted fat lipolysis and oxidation. This effect is prevented by Olfr544 knockdown models both in vitro and in vivo, with supporting bioinformatics analysis. Moreover, EUG reversed gut microbiota dysbiosis and enriched two probiotic strains *L. ruteri XR23* and *L. johnsonii XR25*, and oral gavage potently mitigated NAFLD in mice, with the key metabolites 3‐indolepropionic acid (IPA) and 5‐hydroxyindole‐3‐acetic acid (5‐HIAA) inhibiting lipid synthesis. Lower levels of 5‐HIAA and IPA are observed in patients with NAFLD. These results highlight the considerable potential of EUG as an agonist of Olfr544 for treating NAFLD by inhalation.

## Graphical abstract

A natural compound from the Chinese medicinal plant *Syzygium aromaticum*, eugenol, inhaling which can counteract NAFLD by activating the hepatic olfactory receptor olfr544 and enriching *Lactobacillus* sp. to promoting fat oxidation, catabolism and inhibit lipid synthesis, respectively.

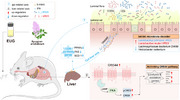

## 1 Introduction

Known as metabolic‐associated fatty liver disease, non‐alcoholic fatty liver disease (NAFLD) affects 20–30% of the population and continues to be the leading cause of chronic liver disease worldwide.^[^ ^1^ ^]^ NAFLD is characterized by hepatic steatosis with insulin resistance, which can induce end‐stage liver disease progression, such as cirrhosis and hepatocellular carcinoma.^[^ ^2^ ^]^ Hepatic oxidative stress and inflammation are the major drivers of lipid accumulation and impaired insulin signaling.^[^ ^3^ ^]^ NAFLD is also strongly associated with metabolic syndrome, obesity, type 2 diabetes, and abnormal lipidemia.^[^ ^4^ ^]^ Strategies to prevent NAFLD are urgently needed due to the limited number of Food and Drug Administration‐approved drugs for treating NAFLD.^[^ ^5^, ^6^ ^]^ Volatile oils (VOs) are volatile and aromatic organic compounds from various parts of plants, such as flowers, leaves, stems, and roots.^[^ ^7^ ^]^ Aromatic Chinese medicine has been reported to include many VOs with antibacterial, antioxidant, anticancer, and hypolipidemic pharmacological activities.^[^ ^8^ ^]^ Increasing evidence indicates that nasal administration can be a route for systemic drug therapy because it ensures rapid drug absorption and is conducive to improving bioavailability.^[^ ^9^ ^]^ Although the nasal administration of VOs is commonly employed to address mood‐related conditions, studies on their use to treat long‐term chronic conditions and their mechanisms remain to be elucidated.^[^ ^10^ ^]^

As the largest multigene family of class A rhodopsin‐like G protein‐coupled receptors, olfactory receptors (ORs) are not only predominantly expressed in the cilia of olfactory sensory neurons, but also ectopically in non‐olfactory tissues, including the testicles, lungs, liver, and adipose tissue.^[^ ^11^, ^12^ ^]^ As more researchers begin to investigate extra‐nasal olfactory receptors, drug developers are noting their therapeutic potential. Recent studies have revealed that ectopic ORs can regulate a variety of metabolic events, including insulin secretion, glucagon secretion, fatty acid oxidation, lipogenesis, and thermogenesis.^[^ ^13^ ^]^ The activation of Olfr544, a hepatic ectopic olfactory receptor, was reported to induce lipolysis in white adipose tissue and fatty acid oxidation in the liver.^[^ ^14^ ^]^ Therefore, we speculate that agonists targeting Olfr544 may become a focal point of ongoing research and development efforts for treating NAFLD.

In a previous study, we found that the VO from *Syzygium aromaticum* (SAVO), a Chinese medicinal plant, could significantly activate Olfr544 in hepatic cells in vitro using high‐throughput screening in our laboratory's existing VOs database; however, whether it can suppress NAFLD is unknown. Here, we investigated the effect and mechanisms of inhaling SAVO on the evolution and deterioration of NAFLD. We demonstrated that a key component of SAVO, eugenol (EUG), alleviated NAFLD development in mouse and cell models better than silybin, which is often used for treating acute and chronic hepatitis and fatty liver. RNA interference analysis determined that EUG treatment activated the ectopic olfactory receptor Olfr544 in the liver and its downstream cAMP/PKA/CREB signaling pathway, thereby promoting fat oxidation and decomposition. Moreover, we also investigated the changes in gut microbiota and discovered the restored gut microbiome. *L. johnsonii XR25* or *L. reuteri XR23* enriched have a negative association with phenotypic markers of NAFLD, transplantation of either can effectively suppress NAFLD in mice, with the key metabolite IPA or 5‐HIAA inhibiting lipid synthesis. Collectively, our study reports a new and promising medicinal VO and its key component, EUG, which can be inhaled to prevent and treat NAFLD through a dual mechanism.

## 2 Results

### 2.1 Inhaling SAVO Inhibits the Development of NAFLD in High‐Fat Diet‐Fed Mice

**Figure 1:**
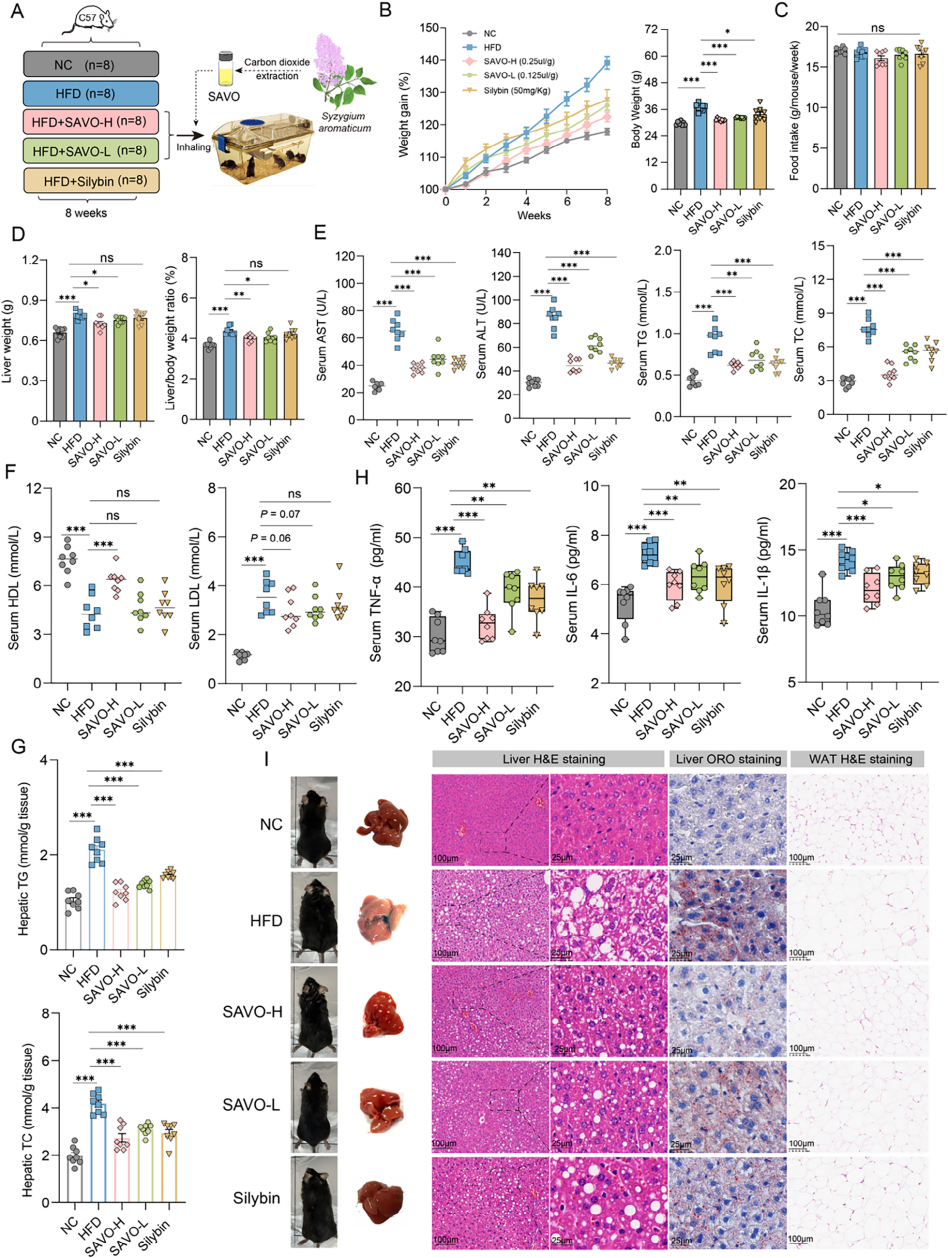
SAVO reduces hepatic steatosis in HFD‐fed mice. A) Experimental outline. C57BL/6 J mice were randomly divided into the NC, HFD, silybin, and SAVO‐treated groups (n = 8/group). B) Weight gain rate (grams). C) Food intake was measured once a week. D) Liver weight and the ratio of liver weight to body weight. E) Serum AST, ALT, TG, and TC contents. F) Serum HDL and HDL contents. G) Hepatic TG and TC contents. H) Serum inflammatory factors. I) Representative H&E and Oil Red O staining images of liver sections (original magnification 100x and 200x). B–H, Statistical analysis was performed using one‐way analysis of variance (ANOVA) followed by Dunnett's test. The data are shown as means ± SEMs (n = 8). ^*^ *p* < 0.05, ^**^ *p* < 0.01. NC, negative control; HFD, high‐fat diet; SAVO‐H, high dose of *Syzygium aromaticum* volatile oil (2.5 µL/10 g); SAVO‐L, high dose of *Syzygium aromaticum* volatile oil (1.25 µL/10 g). TNF‐α, tumor necrosis factor‐alpha; IL‐6, interleukin‐6; IL‐1β, interleukin‐1 beta.

Fatty liver is a metabolic disease caused by abnormal fat metabolism with an excessive accumulation of triglycerides, which can be induced by a persistent high‐fat diet (HFD).^[^ ^15^ ^]^ Mice were fed an HFD and inhaled SAVO for 8 consecutive weeks to evaluate the preventive effect of SAVO on NAFLD (**Figure** **1**A). HFD‐feeding induced typical pathological features of NAFLD, including hepatic steatosis, body fat accumulation, and significant weight gain. Both high and low doses of inhaled SAVO markedly attenuated body weight gain (12.66% and 9.39% lower than the HFD group, respectively, *p*‐value < 0.001), liver weight, and the liver‐to‐body weight ratio in HFD‐fed mice, and was more effective than silybin (Figure 1B,C), a traditional Chinese medicine component commonly used in clinical practice to treat hepatitis and fatty liver. No significant differences in food intake were observed among the groups (Figure 1D), indicating its safety for long‐term inhalation. Excessive dietary fat led to hepatic steatosis, which in turn triggered oxidative stress and inflammation in hepatocytes. SAVO largely alleviated HFD‐induced hepatic steatosis, as evidenced by reduced hepatic triacylglycerol (TG) and total cholesterol (TC) levels, and reduced aspartate transferase (AST), alanine transaminase (ALT), TG, and TC levels in serum. In addition, while only a high dose of SAVO significantly increased the high‐density lipoprotein (HDL) content, both doses suppressed serum low‐density lipoprotein (LDL) levels (Figure 1 E–G). The reversal of changes in pro‐inflammatory cytokines, tumor necrosis factor (TNF)‐α, interleukin (IL)‐6, and IL‐1β in a dose‐dependent manner suggests that SAVO has the potential to repair hepatic inflammation (Figure 1H). Consistent with these findings, hematoxylin and eosin (H&E) and Oil Red O (ORO) staining revealed that the pathological lesions of massive accumulations of lipid droplets in the liver, distorted hepatic lobules, inflammatory cell infiltration, and ballooning caused by HFD feeding could be alleviated by SAVO intervention, with the high‐dose group showing a better effect (Figure 1I). Taken together, these results indicate that SAVO can prevent the onset of diet‐induced NAFLD and its associated metabolic disorders.

### 2.2 SAVO and EUG Promotes Lipolysis by Selectively Activating Olfr544

**Figure 2:**
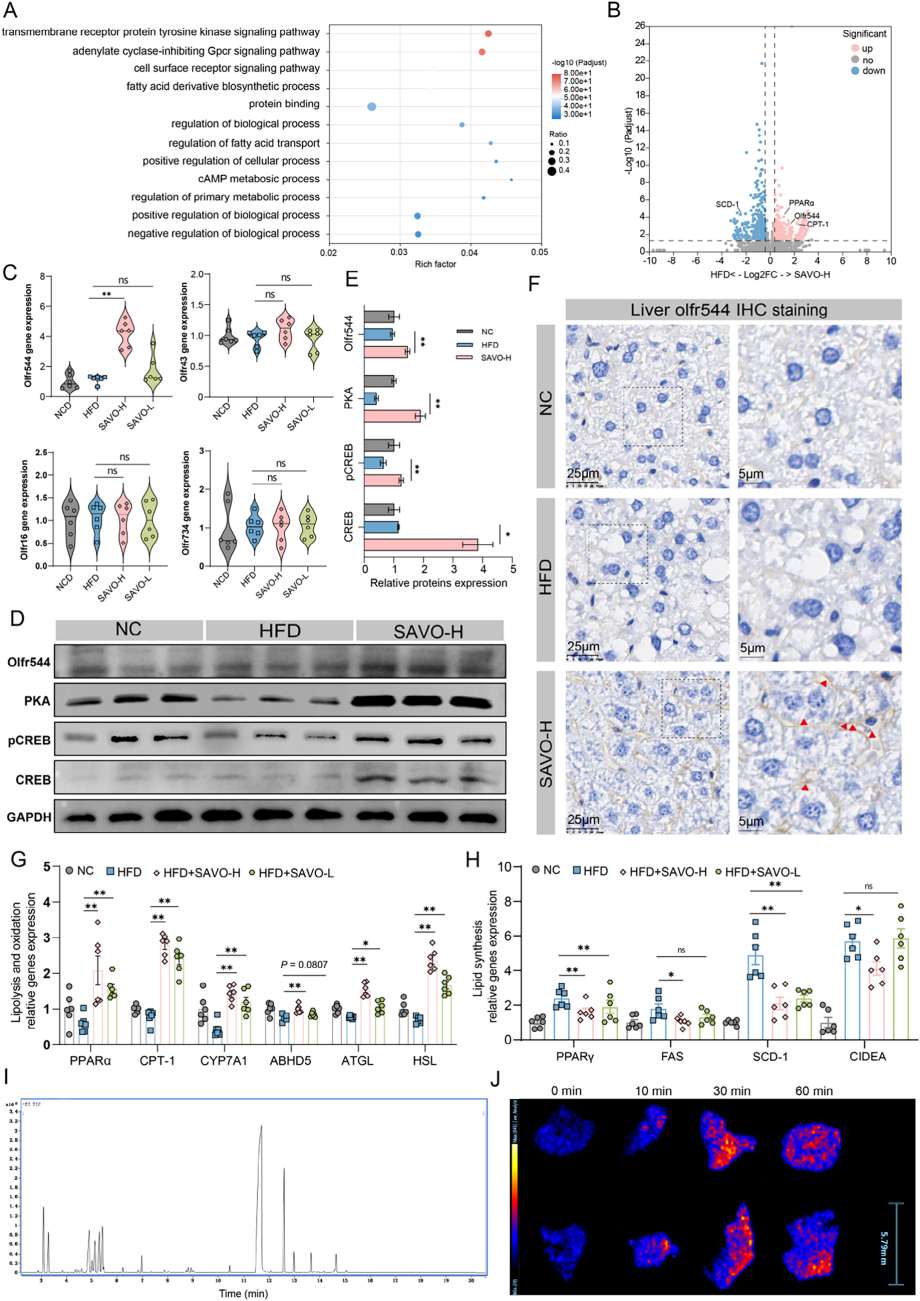
Mechanisms of NAFLD suppression by SAVO in mice. A) GO term analysis of all differentially expressed genes based on RNA‐sequencing data. B) Volcano plot analysis of all differentially expressed genes. Pparγ, Olfr544, CPT‐1, and SCD‐1 were labeled. C) mRNA expression of olfactory receptors in the liver (n = 6). D) Representative immunoblotting of Olfr544, PKA, CREB, and pCREB in livers from different groups expressed as fold‐change relative to GAPDH. E) Relative quantification of (D). F) Liver Olfr544 immunohistochemical staining. The red arrow indicates Olfr544 on the cell membrane. Scale bars: 5 and 25 µm. G,H) Relative gene expression in the livers (n = 6). I) GC‐MS total ion flow diagram of volatile components contained in SAVO. J) Mass spectrometry imaging revealed the EUG content in mouse livers at different time points following SAVO inhalation, where EUG is the primary component of SAVO. Data are presented as means ± SEMs (n = 8). ^*^ *p* < 0.05, ^**^ *p* < 0.01, ns, not significant. NC, negative control; HFD, high‐fat diet; SAVO‐H, high dose of *Syzygium aromaticum* volatile oil (2.5 µL/10 g); SAVO‐L, high dose of *Syzygium aromaticum* volatile oil (1.25 µL/10 g).

Besides transmitting real‐time sensory signals to the brain, ectopic ORs expressed in multiple extra‐nasal and extra‐oral tissues have been reported to be implicated in a variety of processes, including inflammation, adipogenesis, and appetite and energy regulation.^[^ ^16^, ^17^, ^18^, ^19^ ^]^ We performed unbiased RNA sequencing and measured the expression of six metabolism‐related ORs to confirm whether SAVO treatment activated ectopic Olfr544 located in the liver and explored the underlying mechanisms. Gene Ontology (GO) term analysis of differentially expressed genes revealed associations with the adenylated cyclase‐inhibiting G protein‐coupled receptor signaling pathway, the cell surface receptor signaling pathway, and the G protein‐coupled receptor signaling pathway (**Figure** **2**A). Volcano plot analysis of all differentially expressed genes confirmed the significant enrichment of dysregulated genes involved in the regulation of lipolysis, fat digestion, and absorption (Figure 2B). Notably, SAVO treatment selectively and significantly up‐regulated the expression of Olfr544 mRNA in the liver of HFD‐fed mice, without affecting other receptors such as Olfr43, Olfr734, Olfr767, Olfr107, or Olfr16 (Figure 2C; Figure S1A, Supporting Information). Meanwhile, increased Olfr544 protein was substantiated by Western blot (WB) and immunohistochemical (IHC) analyses (Figure 2D–F).

Ligand molecules bind to ORs, and the signals are transmitted downstream through the cyclic adenosine monophosphate (cAMP) signaling pathway. Subsequently, gene transcription is regulated by the activation of CAMP‐dependent protein kinase A (PKA) and the phosphorylation of the cAMP response element binding protein (p‐CREB).^[^ ^20^ ^]^ Previous studies revealed that the cAMP/PKA/CREB signaling axis is involved in fat metabolism in the liver.^[^ ^21^ ^]^ We next verified the changes in levels of related proteins by WB. There was an obvious up‐regulation of PKA, CREB, and p‐CREB proteins in the hepatocytes of SAVO‐treated mice compared to the HFD group (Figure 2D,E). Given the reductions in TC and TG, we hypothesized that SAVO would affect lipid metabolism. We demonstrated that the administration of SAVO increased the mRNA expression of markers of lipolysis and oxidation (carnitine palmitoyltransferase‐1, encoded by CPT‐1; peroxisome proliferator‐activated receptor‐alpha, encoded by PPARα; cholesterol 7 alpha‐hydroxylase, encoded by CYP7A1; adipose triglyceride lipase, encoded by ATGL; hormone‐sensitive triglyceride lipase, encoded by HSL; and abhydrolase domain‐containing protein 5, encoded by ABHD5) in the liver of NAFLD mice, whereas it down‐regulated lipogenesis markers (peroxisome proliferator‐activated receptor γ, encoded by PPARγ; fatty acid synthase, encoded by FAS; stearoyl‐CoA desaturase‐1, encoded by SCD‐1, and cell death‐induced DFFA‐like effector protein A, encoded by CIDEA) (Figure 2G,H).

The chemical constituents of SAVO were analyzed using gas chromatography‐mass spectrometry (GC‐MS) to explore the mechanism of SAVO in more detail. The components of SAVO were identified, and baseline separation was obtained under optimal chromatographic conditions. We selected the first eight components that comprised more than 90% of SAVO for the next verification of whether they activated the Olfr544 receptor (Figure 2I; Figure S1A and Table S3, Supporting Information). We focused on EUG, the predominant component in SAVO. Following SAVO inhalation in mice, the distribution of EUG in hepatic tissues and its serum concentration were analyzed using air flow‐assisted desorption electrospray ionization mass spectrometry imaging (AFADESI‐MSI) and GC‐MS, respectively. The results revealed that EUG levels in serum and liver tissues peaked at 30 min post‐inhalation, demonstrating that volatile components in SAVO could enter the systemic circulation and hepatic tissue following inhalation (Figure 2J; Figure S1B, Supporting Information).

**Figure 3:**
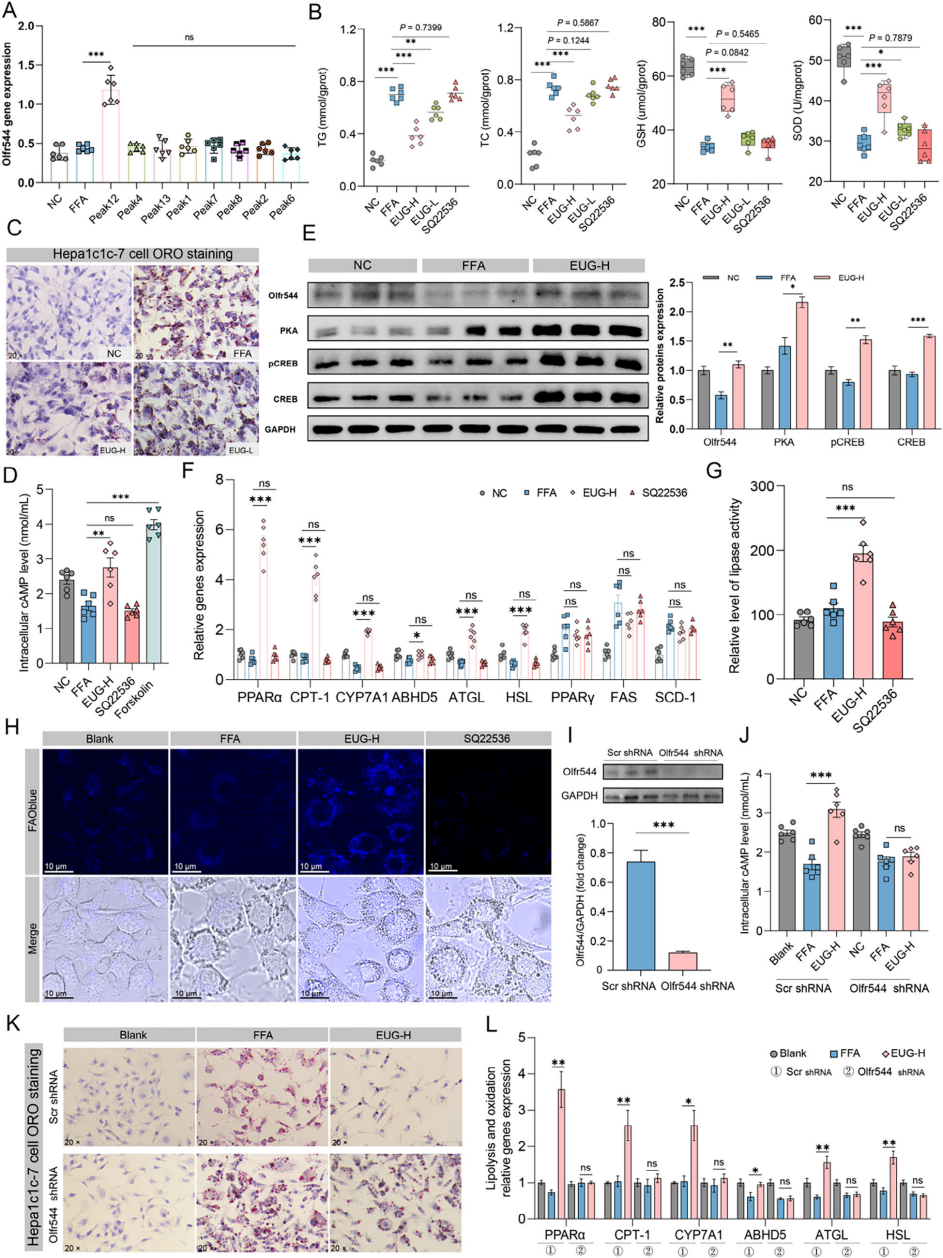
Olfr544 activation is crucial for EUG to reduce FFA‐induced lipid deposition in Hepa1c1c‐7 cells. A) Intracellular Olfr544 gene expression after treatment with different components. B) Intracellular TG, TC, GSH, and SOD levels. C) Hepa1c1c‐7 cell ORO staining (original magnification 200). D) Intracellular cAMP levels in Hepa1c1c‐7 cells were determined by ELISA. SQ22536 is an inhibitor of adenylyl cyclase, and forskolin is an activator of adenylyl cyclase. E) Representative immunoblotting of Olfr544, PKA, CREB, and pCREB in Hepa1c1c‐7 cells. F) Relative gene expression in Hepa1c1c‐7 cells. G,H) Measurement of lipolysis (G) and FAO activity (H) in Hepa1c1c‐7 cells. I) Olfr544 knockdown in Hepa1c1c‐7 cells transfected with shRNA against Olfr544. The protein level of Olfr544 was determined by immunoblotting. Scr, scrambled shRNA. J) Intracellular cAMP levels in Hepa1c1c‐7 cells. K) Hepa1c1c‐7 cell ORO staining (original magnification 20). L) Relative gene expression in Hepa1c1c‐7 cells with Olfr544 gene knockdown. Data are presented as means ± SEMs (n = 6). ^*^ *p* < 0.05, ^**^ *p* < 0.01, ns, not significant. NC, negative control; FFA, free fatty acid; EUG‐H, high‐dose EUG (50 µM); EUG‐L, low‐dose EUG (25 µM).

A free fatty acid (FFA)‐induced lipid accumulation model was established in Hepa1c1c‐7 cells to identify the active compound that can activate Olfr544. Significantly elevated TG levels were observed in cells induced by FFA at concentrations above 0.25 mM (Figure S1C,D, Supporting Information). Based on these results, we chose to use 1 mM FFA to induce cellular steatosis that recapitulates the features of NAFLD. Second, we found that only EUG can activate Olfr544 (**Figure** **3**A), and no signs of cell injury were found microscopically after treatment with other components (data not shown). Therefore, we further explored the mechanism of EUG. As shown in Figure 3B, the administration of EUG led to a dose‐dependent decrease in intracellular lipid accumulation (TG and TC) in Hepa1c1c‐7 cells, as determined by ORO staining (Figure 3B,C). Furthermore, in an in vivo experiment on the effect of SAVO, EUG effectively alleviated oxidative stress induced by FFA, which was manifested as a significant increase in glutamyl cysteinyl glycine (GSH) and superoxide dismutase (SOD) levels (Figure 3B). Additionally, we observed a significant increase in intracellular cAMP levels and the expression of Olfr544, as well as the activation of the downstream cAMP/PKA/CREB signaling pathway (Figure 3D,E), which was consistent with the animal experiment results. We also noted that EUG increased the expression of PPARα, CPT‐1, CYP7A1, ATGL, HSL, and ABHD5 mRNA. Lipase activity and fatty acid oxidation (FAO) activity were assessed by the enzymatic quantification of glycerol release and the FAOBlu Fluorescent Fatty Acid Oxidation Detection Kit, respectively. The results demonstrated that EUG intervention significantly enhanced hepatic lipolysis and β‐oxidation (Figure 3G,H). However, it did not affect the expression of PPARγ, FAS, or SCD‐1 (Figure 3F). Notably, pretreatment with the cAMP inhibitor SQ22536 abolished EUG‐mediated decreases in TG and TC levels, as well as its regulatory effects on associated lipid metabolism genes (Figure 3B–F). These findings suggest that EUG reduces intracellular lipid deposition in vitro by promoting lipolysis and oxidation rather than inhibiting lipid synthesis.

To further elucidate the mechanism, we generated Olfr544 knockout Hepa1c1c‐7 cells by lentiviral transfection (Figure 3I; Figure S1F, Supporting Information). We demonstrated that the knockdown of Olfr544 did not increase intracellular cAMP levels after essential oil stimulation, and the beneficial effects of EUG on FFA‐induced lipid accumulation in Hepa1c1c‐7 cells were eliminated, as evidenced by non‐statistically significant lower intracellular TG and TC levels and higher GSH levels compared with the model group (Figure S1G, Supporting Information). This was also supported by ORO staining, which showed that EUG treatment no longer inhibited FFA‐induced cellular lipid droplet formation (Figure 3K). Additionally, the qPCR results demonstrated that the effect of EUG intervention on lipolysis and oxidation genes disappeared in Olfr544‐knocked down Hepa1c1c‐7 cells (Figure 3L). Taken together, these findings indicate that Olfr544 plays a significant role in mediating the hepaprotective effects of inhaled EUG.

### 2.3 Olfr544 is a Receptor of EUG

**Figure 4:**
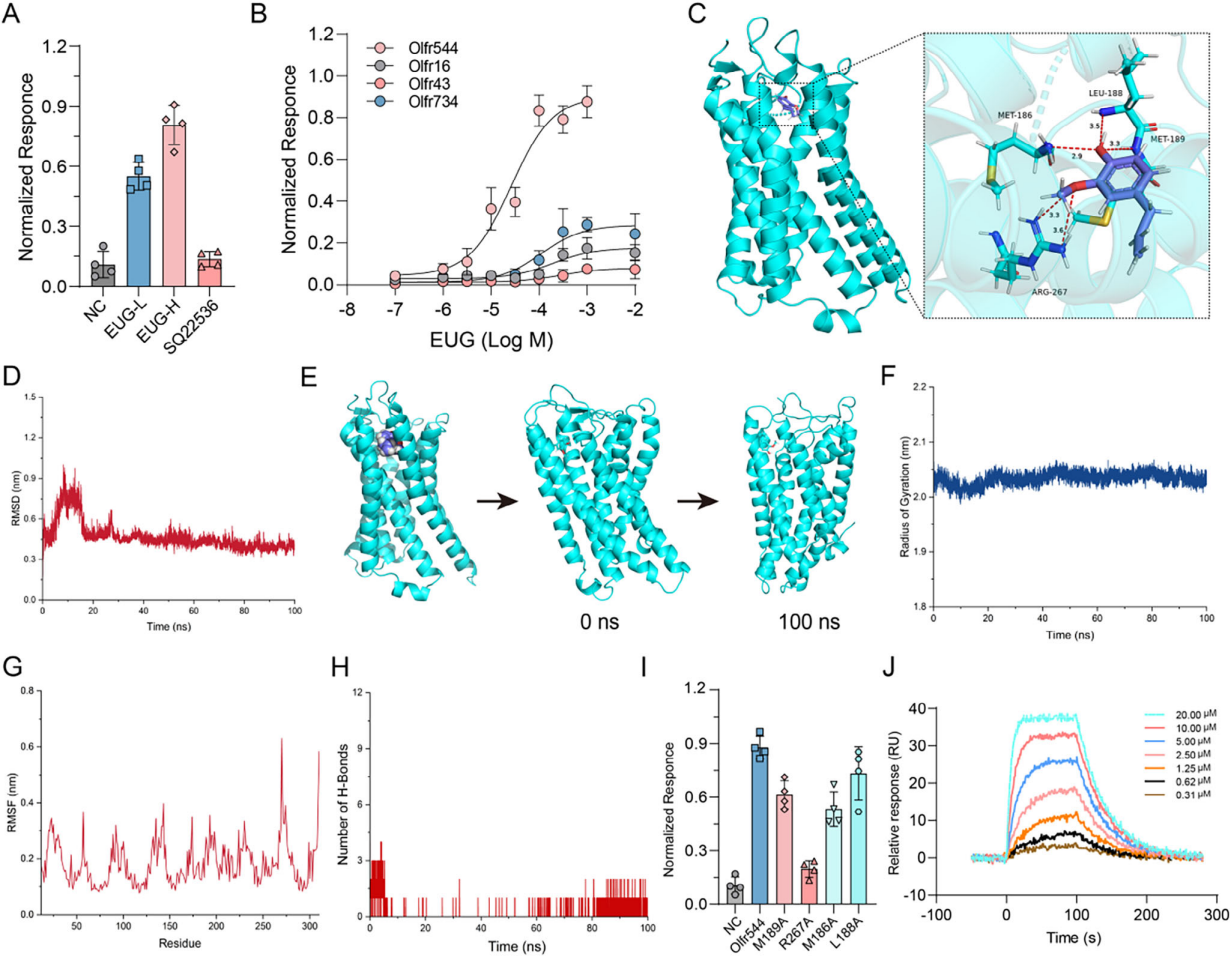
Olfr544 is a receptor of EUG. Bar graphs A) showing the response of Olfr544 to EUG or inhibitor and dose‐response curves of ORs to EUG B). C) Close‐up view of the EUG‐binding site in Olfr544. D) RMSD of EUG‐Olfr544 complexes. E) Morphological changes in EUG‐Olfr544 composite structures during molecular dynamics simulations. F) Rg over the entire simulation. G) RMSF values of the α carbon over the entire simulation. H) Total number of H‐bonds counted over the entire simulation. I) Bar graphs showing the response of Olfr544 and Olfr544 with a point mutation to EUG. J) Dose‐dependent SPR response following EUG injection at varying concentrations. (A, I) The respective highest response value was normalized to 1. Data are presented as means ± SEMs; n = 4.

To further validate the direct binding of Olfr544 to EUG, we conducted cellular functional assays for Olfr544‐expressing Hana3A cells as described previously.^[^ ^22^, ^23^ ^]^ A dose‐dependent increase in normalized luminescence responses to EUG was observed in Olfr544‐expressing cells, and this response was completely abolished by pretreatment with the adenylate cyclase inhibitor SQ22535 (**Figure** **4**A). We also measured the relative responses of multiple olfactory receptors (Olfr734, Olfr43, Olfr16, and Olfr544) to varying concentrations of EUG (Figure 4B). The results confirmed that only Olfr544 exhibited a specific response to EUG.

We observed a dose‐dependent increase in normalized luminescence responses to EUG in Olfr544‐expressing Hana3A cells, which was abolished by the adenylate cyclase inhibitor SQ22535 (Figure 4A). We further measured the relative responses of the olfactory receptors Olfr734, Olfr43, Olfr16, and Olfr544 to varying concentrations of EUG (Figure 4B). Only Olfr544 exhibited a specific response to EUG.

We performed a molecular docking assay to further explore the exact binding sites of EUG and Olfr544 (Figure 4C). The ligand‐protein complexes obtained in the docking studies were used as initial conformations for all‐atom molecular dynamics (MD) simulations. The root mean square deviation (RMSD) curve illustrates the positional stability of a ligand relative to a protein during MD simulations. Analysis revealed significant RMSD fluctuations within the first 40 ns, followed by convergence to a stable trajectory from 40 to 100 ns (Figure 4D). Ligand trajectory analysis confirmed the persistent occupancy of the protein's binding cavity throughout the simulation. Furthermore, structural superimposition of 0 ns (green) and 100 ns (blue) protein‐ligand complexes demonstrated minimal positional deviation (Figure 4E). Analysis of the renormalization group (Rg) trajectory revealed minimal structural deviation, with values oscillating ≈2.05 nm throughout the 100‐ns simulation (Figure 4F). Notably, the Rg curve exhibited further convergence after 80 ns, characterized by reduced fluctuation amplitudes. This stabilization aligns with energy minimization and structural relaxation of the protein‐ligand complex, consistent with the structural convergence observed in RMSD and ligand occupancy analyses.

Root mean square fluctuation (RMSF) analysis was employed to evaluate the structural adaptability and residue‐specific flexibility of the protein during molecular dynamics simulations. The analysis revealed uniformly low RMSF values across all residues, indicating limited conformational fluctuations and stable structural dynamics in the aqueous environment (Figure 4G). Hydrogen bond count analysis between the ligand and protein was conducted to systematically evaluate binding stability throughout the simulation. The number of intermolecular hydrogen bonds fluctuated dynamically during the simulation, stabilizing within a range of 1–2 bonds after 20 ns and persisting until the simulation endpoint (Figure 4H). MM/GBSA binding free energy calculations were performed on the protein‐ligand complex trajectory from 90 to 100 ns after removing periodic boundary effects. Energy decomposition analysis revealed that van der Waals interactions dominated the binding energy (−30.52 kcal mol^−1^), whereas the electrostatic contribution was minimal (−0.17 kcal mol^−1^) (Figure S2A, Supporting Information). This aligns with the hydrogen bond analysis and docking results, where hydrophobic interactions were predominant. The total binding free energy (−33.27 kcal mol^−1^) further confirmed strong binding affinity, corroborating the stability of the EUG‐Olfr544 complex observed in the RMSD and Rg analyses. Collectively, these results support the reliability of the simulation sampling and subsequent mechanistic interpretations, which indicate that EUG may be a favorable ligand for Olfr544 and can form stable complexes.

Alanine‐scanning mutagenesis was performed on key residues (M189, M186, L188, and R267) in Olfr544 to validate the binding residues of Olfr544 predicted by molecular docking and dynamics simulations. Subsequent cellular functional assays demonstrated that alanine substitution at M189, M186, or L188 slightly reduced the response of Olfr544 to EUG, whereas the mutation of R267 to alanine caused a significant decrease in receptor activity (Figure 4I).

To confirm direct binding between EUG and Olfr544, we performed surface SPR – a label‐free, refractive index‐based method monitoring real‐time association kinetics to quantify ligand‐receptor affinity. Given Olfr544's native GPCR structure and the inherent instability of purified GPCRs, we adopted a membrane‐fragment strategy preserving its native conformation in a lipid bilayer.^[^ ^24^, ^25^ ^]^ Accordingly, we isolated membrane fragments from Olfr544‐expressing Hana3a cells using a protocol optimized for olfactory receptors,^[^ ^24^ ^]^ with membrane fragments from untransfected Hana3a cells serving as a negative control. SPR analysis revealed concentration‐dependent responses to EUG on the Olfr544‐expressing membrane‐fragment‐immobilized chip (Figure 4J; Figure S2B, Supporting Information). These results support the reliability of the computational predictions and demonstrate direct binding of EUG to Olfr544.

### 2.4 EUG Resist the Deterioration of NAFLD in HFD‐Fed Mice

**Figure 5:**
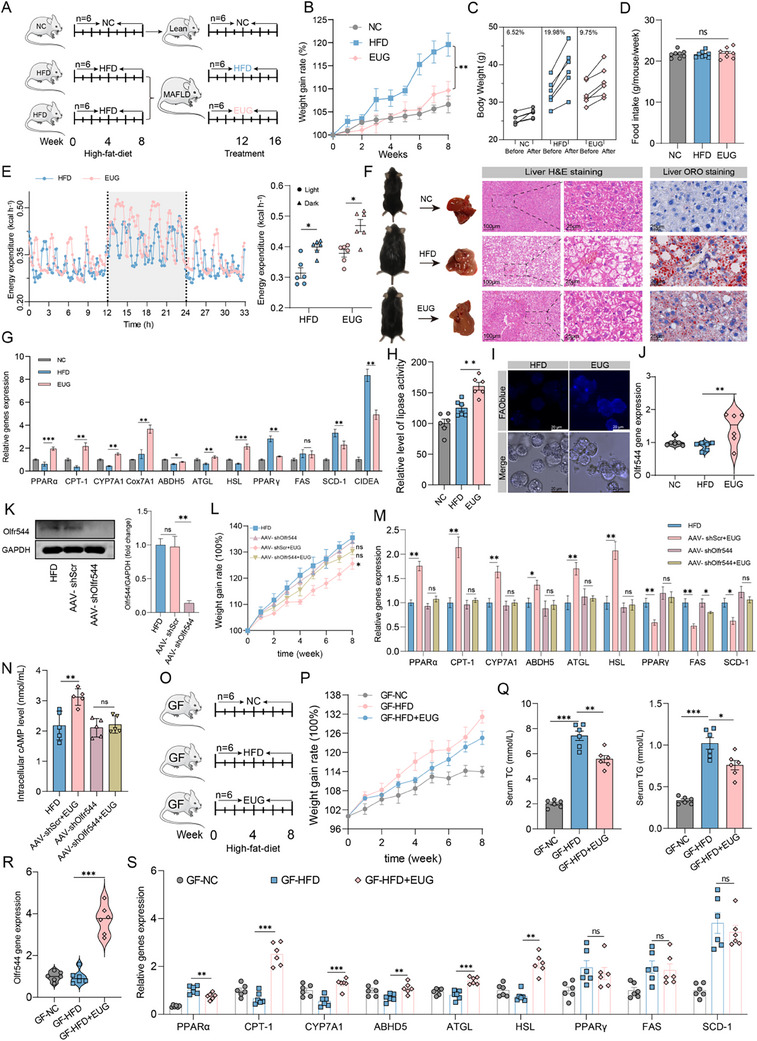
Inhaling EUG suppresses the evolution of NAFLD in HFD‐fed wild‐type, Olfr544 knockdown, and GF mice. A) Experimental outline. WT mice were randomly divided into NC, HFD, and EUG‐treated groups (n = 6/group) after 8 weeks on a high‐fat diet. B) Weight gain rate of mice. C) Changes in body weight of mice during the experimental period (n = 6/group). The numbers represent interval growth rates. D) Food intake was measured once a week. E) Energy expenditure of mice in different groups, measured using an indirect calorimetry system for 33 h. F) Representative H&E and Oil Red O staining images of liver sections (original magnification 20x and 80s). G) Lipid synthesis, lipolysis, and oxidation relative genes expression in the livers. H) Measurement of lipolysis in livers. I) ‌Fatty acid oxidation assay in hepatocytes isolated from mice treated with HFD or EUG. J) Olfr544 mRNA expression in the liver. K) AAV‐8‐TBG‐m‐shOlfr544‐mediated knockdown of Olfr544 in mouse liver. The protein level of Olfr544 was determined by WB. shScr, scrambled shRNA. L) Weight gain rate of mice. M) Lipid synthesis, lipolysis, and oxidation relative genes expression in the livers. N) Intracellular cAMP levels in mouse hepatocytes. O) Experimental outline. GF mice were randomly divided into NC, HFD, and EUG‐treated groups (n = 6/group). P) Weight gain rate of mice. Q) Serum TC and TG levels. R) Olfr544 mRNA expression in the liver. S) Relative expression of lipolysis, oxidation, and lipid synthesis genes in the liver. Data are presented as means ± SEMs (n = 6). ^*^ *p* < 0.05, ^**^ *p* < 0.01, ns, not significant.

We further validated the effect of 8 weeks of inhaling EUG in a new cohort of NAFLD mice after 8 weeks of HFD feeding to verify the therapeutic effects of EUG on NAFLD (**Figure** **5**A). Consistently, EUG treatment resulted in a 10.23% decrease in body weight gain in mice compared to HFD controls over 8 weeks of HFD exposure (Figure 5B,C). EUG treatment did not affect feed intake, whereas it effectively increased energy expenditure, reduced liver steatosis, oxidative stress, and inflammatory damage caused by HFD (Figure 5D–F; Figure S3A—C, Supporting Information). QPCR analysis further confirmed that EUG treatment significantly upregulated hepatic expression of lipolysis‐ and oxidation‐related genes (PPARα, CPT1A, CYP7A1, ATGL, HSL, ABHD5, and Cox7A1) while downregulating lipogenic markers (PPARγ, SCD‐1, CIDEA) (Figure 5G). Functional validation through lipase activity and fatty acid oxidation (FAO) assays demonstrated enhanced hepatic lipolysis and β‐oxidation, consistent with these transcriptional changes (Figure 5H,I). Furthermore, EUG exclusively increased Olfr544 expression without affecting other olfactory receptors in vivo. (Figure 5J; Figure S3D, Supporting Information) These findings suggest that the administration of EUG can prevent the deterioration of diet‐induced NAFLD.

To further validate the necessity of Olfr544 for EUG's function, we constructed AAV‐8‐TBG‐m‐shOlfr544 to achieve liver‐specific knockdown of Olfr544 in mice. As shown in Figure 5K, AAV‐shOlfr544 infection efficiently suppressed endogenous Olfr544 expression, while the scrambled shRNA control group exhibited unaltered Olfr544 levels. Following EUG treatment, Olfr544‐knockdown mice exhibited no reduction in TC/TG levels, and genes governing lipolysis (ATGL, HSL) and fatty acid oxidation (PPARα, CPT‐1) remained unaltered. Notably, among lipogenic genes, FAS decreased significantly while Pparγ and SCD‐1 showed no significant changes (Figure 5L–N; Figure S3E, Supporting Information). To further confirm the necessity of the cAMP‐PKA‐CREB pathway, we performed in vivo pharmacological inhibition using H‐89: co‐administration with EUG abolished TC/TG reduction and blocked EUG‐induced upregulation of lipolysis and β‐oxidation genes (Figure S3F,G, Supporting Information). Collectively, these data demonstrate that Olfr544 is essential for EUG's lipid‐lowering effects and the cAMP‐PKA‐CREB pathway is indispensable for its metabolic actions.

### 2.5 EUG Alters the Gut Microbiota Composition and Enriches *Lactobacillus Johnsonii XR25* and *L. Reuteri XR23*

A strong association between the gut microbiota and the development of NAFLD, non‐alcoholic steatohepatitis, and cirrhosis has been demonstrated.^[^ ^26^ ^]^ Germ‐free (GF) C57Bl/6 mice fed an HFD were treated with EUG by inhalation for 8 weeks, and the relative contribution of the gut microbiota to the anti‐NAFLD effect of EUG was assessed. EUG reduced serum levels of TC and TG and reduced body weight by 6.63% (vs HFD controls, Figure 5O–Q), compared to 9.94% in wild‐type (WT) mice (Figure 5B). QPCR analysis confirmed that EUG significantly upregulated hepatic Olfr544 expression and selectively enhanced the transcription of genes associated with lipolysis and fatty acid oxidation—including PPARα, CPT‐1, ATGL, and HSL, whereas it failed to induce the expression of key lipogenic genes such as PPARγ, FAS, and SCD‐1 in the same model, these demonstrating its direct effect on Olfr544, independent of the microbiota (Figure 5R,S). However, the smaller metabolic improvement in GF mice suggests that the microbiota partially amplifies EUG's efficacy.

**Figure 6:**
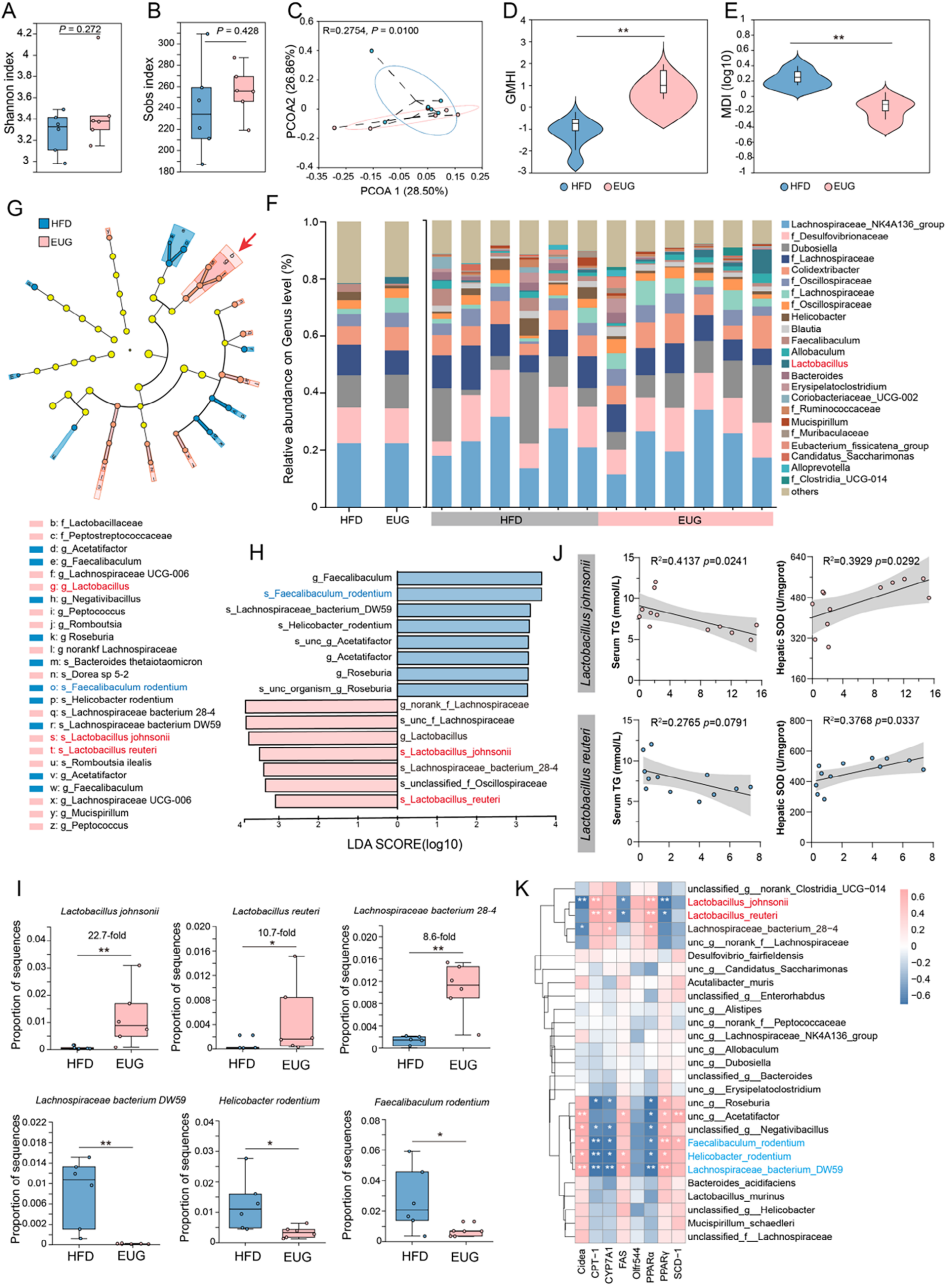
EUG alters the gut microbiota. A) Shannon index and Sobs α‐diversity indexes. B) Sobs index. C) Principal coordinate analysis (PCoA) of all samples by weighted UniFrac distance. (A–C) n = 6/group. D,E) GMHI and MDI of the fecal microbiota. F) Community barplot analysis of the fecal microbiota composition in HFD and EUG‐treated mice at the genus level. G)Analysis of the relative abundance of taxa among different groups using a LEfSe cladogram. Circle sizes in the cladogram plot are proportional to bacterial abundance. From the outer to the inner circle, the circles represent species, genus, class, order, family, and phylum. LDA ≥ 3.0 and *p* ≤ 0.05 (by the Wilcoxon rank‐sum test) were regarded as significant. H) LDA scores representing taxonomic data with significant differences between the two groups. Only LDA scores > 3 are shown. Pink indicates taxa enriched in the EUG group. Blue indicates taxa enriched in the NAFLD group. I) Relative abundance of *Lactobacillus johnsonii*, *Lactobacillus reuteri*, *Lachnospiraceae bacterium 28‐4*, *Lachnospiraceae bacterium DW59*, *Helicobacter rodentium*, and *Faecalibaculum rodentium* in HFD and EUG‐treated groups. J) Correlation analysis of serum TG and hepatic SOD levels with the abundance of *L. reuteri* and *L. johnsonii* in EUG‐treated mice. K) Correlation analysis between clinical factors and metabolites in EUG‐treated mice using partial Spearman's correlation. ^*^ *p* < 0.05, ^**^ *p* < 0.01.

The intestine‐dwelling microbiome composition was investigated using 16S sequencing to determine whether EUG modulates the gut microbiota in alleviating NAFLD. No significant difference in α‐diversity was seen between EUG‐treated and HFD mice, as indicated by Shannon and Sobs index values (**Figure** **6**A,B), whereas PCoA analysis (beta diversity) showed significantly different clustering of gut microbiota in mice in the two groups (Figure 6C). Furthermore, we found that the EUG group had a much higher gut microbiota health index and a lower microbial dysbiosis index, a set of indicators used to assess the ecological health of microorganisms based on species‐level taxonomic characteristics (Figure 6D,E).

In addition, community barplot analysis and linear discriminant analysis (LDA) effect size (LEfSe) analysis revealed that the families *Helicobacteraceae* and *Erysipelotrichaceae*, the genera *Faecalibaculum*, *Acetatifactor*, and *Roseburia*, and the species *Lachnospiraceae bacterium_DW59*, *Faecalibaculum rodentium*, and *Helicobacter rodentium* were enriched in the vehicle group, whereas the *Lactobacillaceae* family, the *Lactobacillus* genus, and the species *L. johnsonii, L. reuteri* and *Lachnospiraceae_bacterium_28‐4* were increased in the gut of EUG‐treated HFD‐fed mice (Figure 6F–H; Figure S4A,B, Supporting Information). At the species level, *L. johnsonii, L. reuteri*, and *Lachnospiraceae_ bacterium_ 28‐4* were enriched 22.7‐fold, 10.7‐fold, and 8.6‐fold, respectively, by EUG treatment. These bacteria have been reported to ameliorate metabolic syndrome and obesity^[^ ^27^, ^28^, ^29^ ^]^ and, thus, may be major contributors to the phenotypic changes in the phenotypic factors of NAFLD by EUG (Figure 6I). The subsequent correlation analysis revealed that the abundance of *L. johnsonii* and *L. reuteri* was negatively correlated with TG, LDL, and MDA levels, and positively correlated with GSH and SOD levels (Figure 6J; Figure S4C,D, Supporting Information). Although the previous experiments in hepatocyte cell models showed that EUG did not affect the expression of lipid synthesis genes, whether this was related to enriched bacteria remains to be revealed. We further performed correlation analysis between bacteria and hepatic gene expression using partial Spearman correlation. We observed that *L. johnsonii* and *L. reuteri* were negatively correlated with lipogenesis (PPARγ, FAS, and Cidea), whereas *Faecalibaculum rodentium* and *Helicobacter rodentium*, which were depleted in EUG‐exposed mice, showed positive correlations (Figure 6K). Collectively, the findings indicate that EUG exerts a protective effect on the liver by altering the gut microbiome, and the insightful correlations provide a strong rationale for further investigations into the potential health benefits associated with these enriched bacteria.

**Figure 7:**
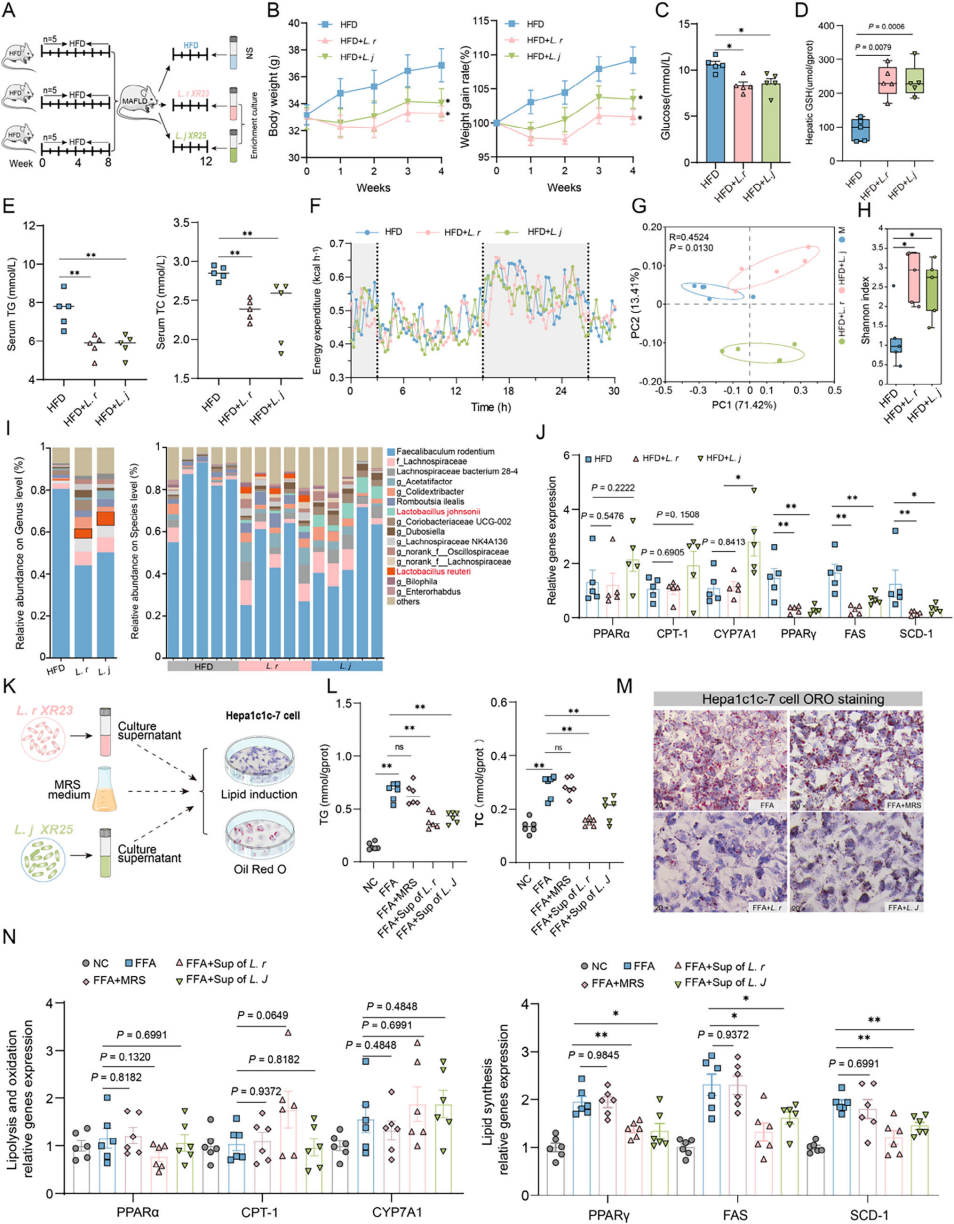
EUG‐enriched *L. reuteri XR23* and *L. johnsonii XR25* ameliorate HFD‐induced NAFLD in mice. A) Schematic of the animal experiment. C57BL/6 J mice were randomly divided into NC, HFD, and bacteria‐treated groups after 8 weeks on a high‐fat diet. Bacteria were enriched, cultured, and then administered to mice by oral gavage. NS represents normal saline. B) Grams of weight gain measured over time and the weight gain rate. C) Fasting blood glucose was measured using a blood glucose dipstick. D) Hepatic GSH content. E) Serum TG and TC contents. F) Energy expenditure of mice in different groups, measured using an indirect calorimetry system for 30 h. G) PCoA of fecal microbiota in mice of different groups. H) Shannon α‐diversity index values. I) Fecal microbiota composition in different groups of mice at the genus and species levels. J) Relative gene expression in the livers. K) Schematic of Hepac1c‐7 cells treated with bacterial supernatants. L) Intracellular TG and TC levels. M) ORO staining pf Hepa1c1c‐7 cells (original magnification 20x). N) Relative gene expression in different treatment groups of Hepa1c1c‐7 cells. Data are presented as means ± SEMs. (A–E, H–J), n = 5/group; (K,L, N), n = 6/group. ^*^ *p* < 0.05, ^**^ *p* < 0.01, ns, not significant.

Considering that compositional shifts in the gut microbiota may play a potential causal role during EUG treatment, we adopted single bacterial transplantation to clarify whether the gut microbiota contributed to NAFLD rescue. We isolated and cultured the enriched bacteria (*L. ruteri XR23* and *L. johnsonii XR25*) from the stool of mice treated with EUG. The bacteria were cultured and prepared as a 2 × 10^8 CFU/0.2 mL solution, followed by transplantation into NAFLD mice via oral gavage (**Figure** **7**A). After 4 weeks, the administration of *L. reuteri XR23* and *L. johnsonii XR25* caused 8.31% and 5.68% weight reductions in the recipient mice compared with HFD control mice, respectively (Figure 7B). We observed that *L. reuteri XR23* and *L. johnsonii XR25* effectively reduced the weight of epididymal, inguinal, and perirenal fat, and blood glucose levels (Figure 7C; Figure S4E, Supporting Information), indicating its potency in regulating blood glucose. Furthermore, both *L. reuteri XR23* and *L. johnsonii XR25* interventions decreased serum AST, TG, and TC levels and hepatic TG levels and increased GSH and HDL‐c content in NAFLD mice. Gavaging *L. reuteri XR23* also significantly lowered the hepatic TC content (Figure 7D,E; Figure S4F,G, Supporting Information). H&E and ORO staining further confirmed the ameliorating effect of *L. reuteri XR23* and *L. johnsonii XR25* treatment on liver fat deposition, in which *L. reuteri* showed a stronger effect (Figure S4I, Supporting Information). However, there was no difference in the energy expenditure of mice in the different groups (Figure 7F).

Intestinal probiotics are often used for the in situ treatment of diseases, such as metabolic disorders, tumors, and chronic inflammatory infections.^[^ ^30^ ^]^ We performed 16S sequencing again to evaluate the probiotic properties of *L. reuteri XR23* and *L. johnsonii XR25*. As shown in Figure 6G,H, the microbiomes in *L. reuteri XR23* or *L. johnsonii XR25*‐treated and HFD‐fed mice were significantly different in terms of alpha and beta diversity. We observed the apparent colonization of *L. johnsonii XR25* and *L. reuteri XR23* and reduced intestinal pathogens, thus confirming a restored microecosystem (Figure 7H,I). RNA sequencing validated that *L. reuteri XR23* and *L. johnsonii XR25* administration did not regulate the expression of hepatic Olfr544, which indicates that the EUG activation of Olfr544 is independent of bacteria (Figure S4H, Supporting Information). We also found no significant changes in key genes related to fatty acid oxidation (PPARa/CPT‐1) or cholesterolysis (CYP7A1). However, there was a considerable decrease in the levels of PPARγ, FAS, and SCD‐1 mRNAs involved in regulating lipid synthesis (Figure 7J). Therefore, we conjectured that the inhibition of lipid synthesis by EUG may be due to the increased abundance of *L. reuteri XR23* and *L. johnsonii XR25* rather than a direct effect of EUG.

### 2.6 The Key Active Microbial Metabolites IPA and 5‐HIAA can Ameliorate NAFLD

Having shown that EUG protects against NAFLD by modulating the gut microbiota, we next sought to investigate the function of EUG‐enriched probiotics, *L. reuteri XR23* and *L. johnsonii XR25*, in NAFLD and determine the role of some metabolites. Consistent with our previous hypothesis, TC and TG levels were significantly suppressed in FFA‐induced Hepa1c1c‐7 steatosis cells treated with the culture supernatants of *L. reuteri XR23* or *L. johnsonii XR25*, but not MRS medium (Figure 7K,L). This was further confirmed by OAO staining, which showed that FFA‐induced lipid droplet accumulation in Hepa1c1c‐7 cells was significantly reduced (Figure 7M). In the in vitro experiment, treatment with *L. johnsonii XR25* and *L. reuteri XR23* supernatants effectively downregulated the expression of lipid synthesis genes PPARγ, FAS, and SCD‐1 (Figure 7N).

**Figure 8:**
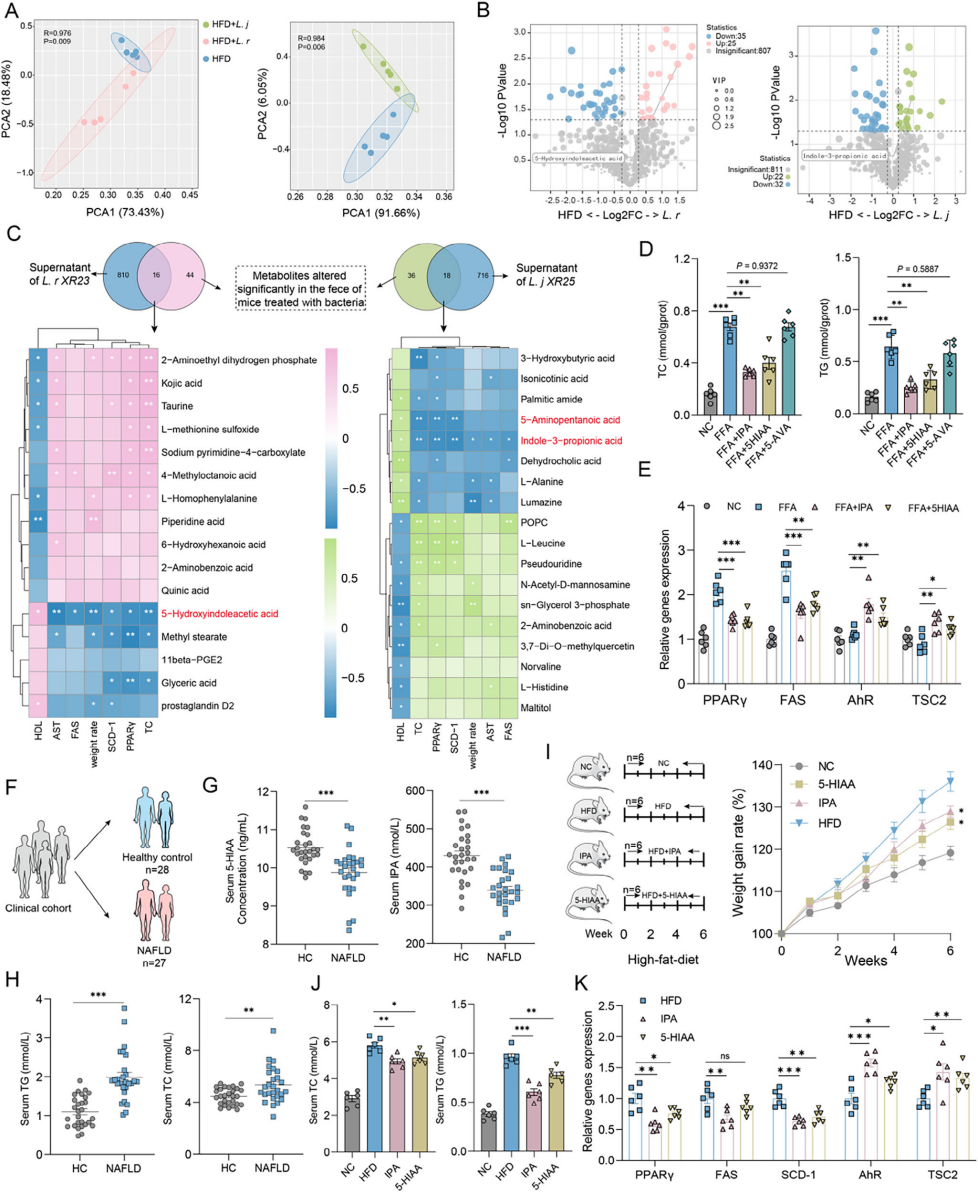
The microbial metabolites IPA and 5‐HIAA can ameliorate NAFLD. A) Principal components analysis of fecal metabolites in HFD and bacteria‐treated mice (n = 5). Differences in beta diversity were evaluated by analysis of similarity (ANOSIM). B) Volcano plots showing altered fecal metabolites in the HFD + *L. j* group and the HFD + *L. r* group compared to the HFD group. (n = 5 per group; fold‐change < −1.3 or >1.3, *p* < 0.05). C) Correlation analysis between clinical factors and metabolites using partial Spearman's correlation. D) Intracellular TG and TC contents. E) Relative gene expression in Hepa1c1c‐7 cells. F) Subjects with and without NAFLD were recruited. G) Serum concentrations of 5‐HIAA and IPA in individuals (HC, healthy control, n = 28; NAFLD patients, n = 27). H) Schematic of the animal experiment. Mice were randomly divided into NC, HFD, and IPA or 5‐HIAA‐treated groups. I) Weight gain rate measured over time. J) Serum TG, TC, AST, and ALT contents. K) Relative gene expression in different treatment groups of mice. Statistical analyses were performed using a two‐tailed Wilcoxon test, and the data are presented as means ± SEMs. ^*^ *p* < 0.05, ^**^ *p* < 0.01.

Non‐targeted metabolomics analysis was conducted with mouse feces to profile the metabolic changes. PCA of the overall metabolite composition revealed that each bacterial treatment group was significantly separated from the HFD group (**Figure** **8**A), indicating that *L. johnsonii XR25* and *L. reuteri XR23* treatment altered the gut metabolite profiles. Similarly, the volcano plot showed that ≈20 metabolites were up‐regulated and ≈30 were down‐regulated after bacterial treatment (Figure 8B; Figure S5A, Supporting Information). We next sought to identify the metabolites derived from bacterial treatment. As the supernatants decreased TC and TG levels in hepatocytes and suppressed lipid synthesis in vitro, we speculated that some functional metabolites might be shared and significantly altered in mouse fecal contents after bacterial treatment and in their culture supernatants. Thus, we took the intersection of the total metabolites in the bacterial supernatant with the metabolites that were significantly altered in the feces of mice treated with the bacteria relative to those in the NAFLD group and performed a correlation analysis to determine the potential association between the shared metabolites and clinical factors. We found that the 5‐hydroxyindoleacetic acid (5‐HIAA) metabolites up‐regulated after *L. reuteri XR23* treatment and the indole‐3‐propionic acid (IPA) metabolites up‐regulated after *L. johnsonii XR25* treatment were significantly negatively correlated with weight rate, TC, AST, FAS, SCD‐1, and PPARγ, and positively correlated with HDL (Figure 8C). In addition, the correlation analysis between the altered TOP 10 bacteria and metabolites showed that the abundance of *L. johnsonii XR25* and *L. reuteri XR23* was positively correlated with the IPA and 5‐HIAA contents, respectively, implying that *L. johnsonii XR25* and *L. reuteri XR23* treatment could promote the formation of IPA and 5‐HIAA, which might be achieved by colonization of the intestinal tract of mice and the promotion of the co‐generation of other bacteria in the genera *Coriobacteriaceae UCG‐002* and *Lachnospiraceae NK4A136* (Figure S5B,C, Supporting Information). Hence, *L. johnsonii* and *L. reuteri* enriched by EUG could modulate metabolites to resist NAFLD.

We repeated the cell experiments with three metabolites, IPA, 5‐HIAA, and 5‐aminovaleric acid (5‐AVA), another metabolite that was negatively correlated with TC, SCD‐1, and PPARγ to study whether these metabolites directly suppressed NAFLD. IPA and 5‐HIAA, but not 5‐AVA, reduced TG and TC levels in FFA‐induced Hepa1c1c‐7 cells and suppressed the expression of lipid synthesis genes, including PPARγ and FAS (Figure 8D,E). Since 5‐HIAA and IPA are established agonists of the aryl hydrocarbon receptor (AHR),^[^ ^31^, ^32^ ^]^ we further measured AHR activity in treated Hepa1c1c‐7 cells and observed a significant up‐regulation of AHR (Figure 8E). Mechanistically, AHR activation promotes its binding to the TSC2 gene promoter, enhancing TSC2 expression,^[^ ^33^ ^]^ which, in turn, suppresses PPARγ‐mediated lipogenesis^[^ ^34^, ^35^ ^]^ These findings align with our experimental results, supporting that the AHR/TSC2 axis might underlie the anti‐lipogenic effects of 5‐HIAA and IPA.

To explore the physiological significance of these two metabolites in NAFLD development, we examined their amounts in the serum of clinical patients. A total of 55 serum samples were analyzed, among which 27 were diagnosed as having hepatic steatosis. Twenty‐eight were used as healthy controls without hepatic steatosis. The patient information is described in the (Table S1, Supporting Information). The results showed a significant reduction in 5‐HIAA and IPA levels in patients with hepatic steatosis compared with healthy controls, which was accompanied by a significant increase in serum TC and TG levels (Figure 8F–H). This suggests that decreases in 5‐HIAA and IPA might be positively correlated with the occurrence and development of NAFLD.

The therapeutic potential of 5‐HIAA and IPA as novel candidates for NAFLD intervention was evaluated in HFD‐induced NAFLD mice (Figure 8I). As anticipated, both metabolites significantly attenuated HFD‐induced NAFLD progression, evidenced by improved serum profiles: reduced total cholesterol (TC), alanine aminotransferase (ALT), and aspartate aminotransferase (AST) levels (Figure 8J,K). Consistent with prior in vitro findings, 5‐HIAA and IPA selectively upregulated hepatic expression of lipogenic genes (Figure 8J,K; Figure S6, Supporting Information). Taken together, these results suggest that metabolites altered by bacteria after EUG treatment are involved in preventing NAFLD. Altogether, our studies provide in vitro and in vivo evidence that 5‐HIAA and IPA can inhibit the NAFLD process. Decreased circulating 5‐HIAA and IPA levels can be hallmarks of NAFLD.

## 3 Discussion

NAFLD has become a public health issue of global concern; however, there are no currently approved treatment drugs.^[^ ^36^ ^]^ Most of the drugs approved by the Food and Drug Administration to treat metabolic diseases are administered orally or by injection, which has the disadvantage of poor patient compliance. Oral administration is still the traditional way of treating diseases in traditional Chinese medicine, so there is still an urgent need to develop better patient compliance delivery forms.^[^ ^37^ ^]^ Here, we innovatively established a method of NAFLD treatment by inhaling a Chinese herbal volatile oil. In this study, we systematically evaluated the protective effect of SAVO in the evolution of NAFLD in mice and demonstrated that it was more effective than sylibin in alleviating NAFLD progression. Inhaling SAVO or its main component EUG significantly changed the NAFLD phenotype, including reducing the weight of experimental mice, steatosis, and serum lipid levels, increasing the antioxidant capacity, and reducing inflammation. These results indicate that inhaling SAVO or EUG, as an emerging new mode of administration, is a very promising strategy for preventing metabolic diseases compared to conventional oral administration with poor patient compliance in the context of modern high‐fat and high‐calorie diets.

Plant VOs are natural active ingredients with certain functions in regulating lipid metabolism.^[^ ^38^ ^]^ Studies have shown that turmeric, pepper, patchouli, and basil VOs could reduce lipid accumulation and inflammation in NAFLD animal models and enhance lipid metabolism.^[^ ^39^ ^]^ Hong et al. found that inhaling fennel VO improved the antioxidant capacity of mice and reduced liver damage caused by an HFD.^[^ ^40^ ^]^ These findings emphasize VOs as a complementary medicine for NAFLD and provide a foundation for further research and clinical application. *Syzygium aromaticum* is a medicinal and edible plant widely grown in Southeast Asia. It was recorded to have the effects of warming the kidney, strengthening the spleen, and downbearing counterflow in the ancient Chinese medical book *Collected Works of Materia Medica*, and it is often used to improve endocrine and metabolic diseases. Our study revealed the main component of EUG has significant preventive and therapeutic potential against HFD‐induced NAFLD. Specifically, EUG significantly decreased the weight of NAFLD mice while effectively improving liver function and reducing oxidative stress and the inflammatory response without affecting food intake. In vitro experiments further confirmed that EUG intervention alleviated FFA‐induced lipid deposition and oxidative stress in Hepc1c1c‐7 cells, consistent with the facilitation of energy expenditure seen in animal experiments.

Since the discovery of the olfactory receptor family in 1991, increasing evidence has shown that these receptors are widely distributed in non‐olfactory tissues, particularly lipid‐related tissues (liver, fat, and small intestine).^[^ ^41^, ^42^ ^]^ Olfactory receptors related to lipid metabolism play a regulatory role by coupling to various G proteins.^[^ ^43^ ^]^ Other studies have shown that carvone can reduce hepatic steatosis with liver Olfr43 acting as the receptor.^[^ ^44^ ^]^ The activation of Olfr544, an OR expressed in the liver and adipose tissue of mice that regulates cellular energy metabolism and obesity, was reported to exert anti‐obesogenic effects in HFD‐fed mice by regulating inter‐organ energy metabolism.^[^ ^14^ ^]^ Given that VOs are administered by inhalation, we speculate that some VOs may inhibit NAFLD by activating Olfr544. Here, we found and determined that only the expression of olfactory receptor Olfr544 was up‐regulated in the liver after SAVO or EUG treatment, which was confirmed in Hepa1c1c‐7 cells in vitro. In addition, knocking down Olfr544 significantly inhibited the ameliorating effect of EUG on lipid deposition in Hepa1c1c‐7 cells. This protective effect on the liver through olfactory receptors was supported by previous studies reporting that inhaling orange VO improved the antioxidant capacity of mice and reduced liver damage caused by HFD, and the Olfr544 ligand, azelaic acid, specifically induced lipolysis in adipocytes and promoted fatty acid oxidation in the liver.^[^ ^14^, ^45^, ^46^ ^]^ Notably, the liver, responsible for ≈20% of basal metabolic rate,^[^ ^47^ ^]^ critically regulates systemic energy expenditure. Our study revealed that EUG treatment significantly increased hepatic ATGL activity and β‐oxidation. ATGL drives triglyceride hydrolysis, liberating free fatty acids for mitochondrial β‐oxidation‐a process generating ATP and heat that directly elevates energy expenditure.^[^ ^48^ ^]^ As established in prior work, ATGL‐mediated lipolysis is indispensable for hepatic β‐oxidation and mitochondrial energy regulation,^[^ ^49^ ^]^ consistent with our observed EUG‐induced energy expenditure increase.

The ligand‐dependent activation of ‌ORs in non‐olfactory tissues and cells was reported to activate multiple intracellular signal transduction pathways, with cAMP and Ca^2+^ being the two main secondary messengers.^[^ ^50^, ^51^, ^52^ ^]^ In the process of hepatic lipid metabolism, the cAMP signaling pathway can phosphorylate CREB by triggering PKA.^[^ ^53^ ^]^ CREB phosphorylation can affect lipid synthesis, degradation, and transport by regulating downstream gene expression, thereby affecting hepatic lipid metabolism balance.^[^ ^54^ ^]^ Consistent with these findings, EUG intervention was observed to increase cAMP levels and up‐regulate PKA and CREB protein expression both in vitro and in vivo, leading to the phosphorylation of CREB. Notably, these effects were abolished by pretreatment with the cAMP inhibitor SQ22536 in cellular models and the PKA inhibitor H‐89 in mice, demonstrating that the cAMP/PKA/CREB axis mediates EUG's beneficial effects on NAFLD. These results are consistent with previous reports that EUG could induce cAMP and calcium levels in preadipocyte 3T3‐L1 cells^[^ ^55^ ^]^ and that 50 and 100 µM EUG microcapsules could significantly inhibit lipid accumulation and reduce TC and TG levels in steatotic HepG2 cells induced by FFA.^[^ ^56^ ^]^ We performed cellular functional assays to investigate whether EUG directly binds to Olfr544^[^ ^22^, ^23^, ^57^ ^]^ and validate odorant‐OR interactions. In Olfr544‐expressing Hana3A cells, EUG elicited a dose‐dependent increase in normalized luminescence response, which was abolished by the adenylate cyclase inhibitor SQ22536. In contrast, no significant response was observed in cells expressing other olfactory receptors. These functional data, combined with Olfr544 activation‐mediated metabolic changes (enhanced fatty acid oxidation, lipolysis, and reduced hepatic triglyceride accumulation), Western blot validation (PKA/CREB phosphorylation), and gene knockdown experiments (abolished EUG effects in Olfr544 knockdown hepatocytes and mice models), collectively confirm that EUG directly activated Olfr544 to exert lipid‐regulatory effects. Furthermore, the molecular docking assay and MD were performed to investigate stability. Given the strong hydrophobicity and limited hydrogen‐bond acceptors of EUG, the primary interactions with Olfr544 were mediated by hydrophobic forces, which aligns with prior findings of hydrophobic‐driven ligand binding in olfactory receptors, such as propionate binding to OR51E2.^[^ ^12^ ^]^ Structural analysis confirmed that EUG occupies the ligand‐binding cavity of Olfr544, forming multiple non‐covalent interactions with favorable free energy. SPR directly detects ligand‐receptor binding interactions.^[^ ^25^ ^]^ However, since Olfr544 is a GPCR requiring a lipid bilayer to maintain its functional conformation, we employed membrane fragment‐based SPR analysis. This approach demonstrated concentration‐dependent responses to EUG on the Olfr544‐expressing membrane‐fragment‐immobilized SPR chip. These results suggest a stable ligand‐receptor complex. Together, our study findings offer insights into how EUG might suppress NAFLD development through the Olfr544/cAMP/PKA/CREB axis. However, despite employing MD simulations, the absence of structural validation of the EUG‐Olfr544 complex by nuclear magnetic resonance spectroscopy or X‐ray crystallography necessitates that these findings remain predictive and are subject to deviations from the experimental structures.

Evidence of the role of the gut microbiota in various metabolic disorders is accumulating, and the manipulation of which might be a novel therapeutic option. We performed a more in‐depth study than previous studies and confirmed that EUG activated the receptor Olfr544 and further demonstrated that inhaling EUG could inhibit liver lipid synthesis and promote lipolysis in HFD mice, whereas the former effect has not been found in in vitro cell experiments and the GF mice experiment. Moreover, we observed no significant reduction in gene expression mediated by shOlfr544‐induced Olfr544 knockdown in either cellular or animal models. Hence, we speculate that inhibiting hepatic lipid synthesis may not be a direct effect of EUG. Considering the ameliorated NAFLD phenotype (e.g., TC, TG levels) in GF mice treated with EUG, we propose that the gut microbiota contributes to EUG's mechanism of action. In our study, we found that EUG treatment could reduce the abundance of *Faecalibaculum rodentium*, a bacteria that is often positively correlated with HFD consumption.^[^ ^58^, ^59^ ^]^ However, EUG selectively enriched several species in the *Lactobacillus* genus and some *Lachnospiraceae* family bacteria, including *L. johnsonii*, *L. reuteri, and Lachnospiraceae_bacterium_28‐4*. *Lactobacillus* is a widely used traditional probiotic in the treatment of NAFLD. Based on in vitro anaerobic workstations, we isolated and cultured top‐ranked EUG‐alter bacteria, including culturable bacteria *L. johnsonii* and *L. reuteri*. These bacteria have been reported to ameliorate obesity, metabolic syndrome, and inflammation and improve insulin resistance, while their mechanisms have not been fully elucidated.^[^ ^60^, ^61^, ^62^ ^]^ More critically, a significant correlation was observed between *L. johnsonii*, *L. reuteri*, and the clinical phenotype of NAFLD in our research. We sought to functionally validate EUG‐enriched bacteria, demonstrating that *L. johnsonii* and *L. reuteri* transplantation effectively suppressed NAFLD in mice, as evidenced by reductions in hepatic steatosis, inflammation, and serum markers of NAFLD and halted weight gain. Consistent with our observations, a preparation based on *Lactobacillus johnsonii* BS15 has been shown to alleviate diet‐induced obesity and hyperlipidemia by reducing intestinal permeability and down‐regulating liver inflammatory factors.^[^ ^63^ ^]^ We performed 16S sequencing again to determine whether these two strains of bacteria colonized the mouse intestinal tract.

Consistent with previous results, we observed significant decreases in *Faecalibaculum rodentium* and the apparent colonization of *L. johnsonii* and *L. reuteri*, which led to the increasing alpha diversity of the microbiome rather than over‐occupying the ecological niche and creating a dominant imbalance. Therefore, EUG promoted the outgrowth of beneficial commensals and created niches less favorable for bacterial pathogens. However, we observed no significant changes in the growth curves of *L. johnsonii XR25* or *L. reuteri XR23* at varying concentrations of EUG in vitro (data not shown). Therefore, the mechanism by which EUG inhalation increases the abundance of *L. johnsonii* and *L. reuteri* in the intestine requires further study.

Microbial metabolites are crucial mediators of the intestinal flora's effects on host physiological processes.^[^ ^64^, ^65^ ^]^ The in vitro experiments showed that *L. johnsonii XR25* or *L. reuteri XR23* supernatant, but not MRS medium, could decrease lipid deposition and enhance anti‐oxidation in Hepc1c1c‐7 cells. We reasoned that the protective effect of *L. johnsonii XR25* and *L. reuteri XR23* might be attributed to the production of some metabolites. A detailed systematic investigation of the metabolites of each strain was conducted. Untargeted metabolomic analysis of the intersecting differential metabolites in the stool of mice treated with *L. johnsonii XR25* or *L. reuteri XR23* and NAFLD mice treated with the culture supernatant of the corresponding bacteria was performed. We first identified IPA and 5‐HIAA as the key metabolites downstream of *L. johnsonii XR25* and *L. reuteri XR23*, respectively. Like their bacterial supernatants, IPA and 5‐HIAA showed similar abilities to down‐regulate the expression of lipid synthesis genes such as PPARγ and FAS, corresponding to the inhibition of lipid synthesis genes by SAVO or EUG in in vivo experiments. We observed that 5‐HIAA and IPA treatment up‐regulated AHR and TSC2 expression in hepatocytes. Elevated TSC2 inhibits mTORC1 activity, and both metabolites are implicated in regulating lipogenesis via the AHR/TSC2/mTORC1 axis.^[^ ^35^, ^66^ ^]^ These findings provide a mechanistic framework for further investigations of their anti‐lipogenic roles. Given that the transplantation of neither bacteria affected the energy expenditure of the mice, we concluded that the regulation of the gut microbiota by EUG ultimately played a role in inhibiting lipid synthesis. In agreement with our results, some researchers found that IPA treatment of human immortalized hepatocyte cells lowered cholesterol‐induced lipid accumulation in the cells.^[^ ^67^ ^]^ Other studies have shown that the administration of 5‐HIAA improved glucose intolerance and obesity in HFD‐fed mice while preserving hepatic insulin sensitivity.^[^ ^31^ ^]^ However, few studies on the correlation between 5‐HIAA and the progression of NAFLD have been conducted. In recent years, gut microbially produced IPA was reported to inhibit atherosclerosis and boost immunity,^[^ ^68^, ^69^ ^]^ and the beneficial effect of IPA on the liver has been highlighted. More importantly, we determined that microbiota‐dependent 5‐HIAA and IPA were also associated with NAFLD, as patients with NAFLD showed decreased serum concentrations. Further studies in mice proved the potential effects of 5‐HIAA and IPA in improving NAFLD. Whether 5‐HIAA and IPA can be employed as biomarkers for NAFLD diagnosis requires a larger cohort study, including an in‐depth exploration of their mechanisms of action. Taken together, these data suggest that EUG mediates the *L. johnsonii*‐IPA and *L. reuteri‐*5‐HIAA axes, alleviating NAFLD.

## 4 Conclusion

**Figure 9:**
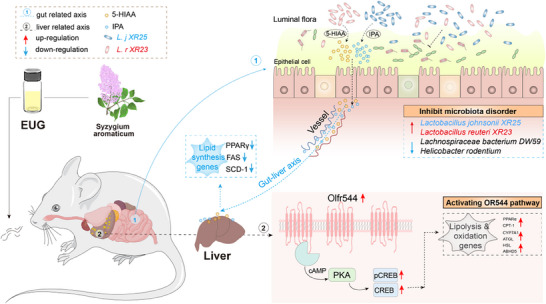
Schematic diagram of the mechanisms of EUG in suppressing NAFLD. On one hand, inhaling eugenol can promote the decomposition and oxidation of liver fat by activating the olfr544‐cAMP‐PKA pathway. On the other hand, the improved gut microbiota, especially the enriched *L. reuteri XR23* and *L. johnsonii XR25* can inhibit the synthesis of lipid by producing 5‐HIAA and IPA, which form a synergistic and complementary effect.

This study demonstrated for the first time that inhaling EUG was effective in suppressing NAFLD by establishing diet‐induced steatohepatitis in mice through dual pathways (**Figure** **9**). Through integrated in vitro and in vivo approaches involving Olfr544 knockdown models, comprehensive cellular functional assays, kinetic simulations, and SPR analysis, we demonstrate that EUG activates hepatic Olfr544 to trigger the cAMP‐PKA‐CREB signaling cascade, thereby enhancing fat oxidation and lipolysis processes. Furthermore, by integrating microbiome sequencing, supplementation with functionally relevant cultured bacteria in animal models, and metabolomic profiling, we demonstrated that gut microbiota remodeling induced by 8‐week EUG treatment significantly contributes to NAFLD amelioration. Subsequently, we validated IPA and 5‐HIAA produced by *L. johnsonii XR25* and *L. reuteri XR23* enriched by EUG treatment, respectively, as hepatoprotective metabolites. These findings suggest EUG as a candidate for counteracting diet‐induced NAFLD by inhalation through a dual mechanism as a novel agonist of the hepatic ectopic olfactory receptor Olfr544. Targeting ectopic olfactory receptors and the gut microbiota may provide new insight and evidence for inhalation therapy to treat metabolic disorders. However, the potential pleiotropic roles of this traditional Chinese medicine VOs in humans still need to be addressed in future research.

## 5 Experimental Section

### Ethics and Human Sample Preparation

All work was approved by the Medical Ethical Committee of the First Affiliated Hospital of Henan University of Chinese Medicine (No.2024HL‐439‐01) (Zhengzhou, Henan, China). Medical records were evaluated and treated by licensed medical professionals to confirm the clinical characteristics of the patients. All blood samples were collected in compliance with the informed consent policy. Clinical information is summarized in Table S1 (Supporting Information).

### Animal Experiments

All animal experimental procedures complied with the regulations of the People's Republic of China on the management of experimental animals and were approved by the Animal Experimentation Ethics Committee of Henan University of Chinese Medicine (No. IACUC‐202310024). Seven‐week‐old C57/BL6J male mice (SPF, Pengyue Biotechnology, Shandong, China) were housed in groups of four to six animals per cage in a pathogen‐free facility with standard bedding and enrichment in a temperature and humidity‐controlled room on a 12 h light/dark cycle, with free access to food and water. After 1 week of recovery, the animals were randomly assigned to the normal diet‐fed control group (NC, n = 8), the high‐fat diet‐fed control group (HFD, n = 8), and the experimental group (HFD+SAVO, n = 8) with different doses of SAVO inhaled for 8 weeks once a day, or EUG treatment for 8 weeks after 8 weeks of continuous HFD modeling (n = 6/group). Briefly, VO was smeared on both sides of the nose alars of the mice. The cages were closed for 1 h with only the aeration holes retained, after which the cages were returned to the individual ventilated caging system. 0.25uL g^−1^ body weight per mice in the high dose group and 0.125uL g^−1^ body weight in the low dose group. Seven‐week‐old C57/BL6J male mice (SPF, Pengyue Biotechnology, Shandong, China) were housed and treated as above, then treated with 30 mg kg^−1^ 5‐HIAA and 15 mg Kg^−1^ IPA i.g., once per day. Food intake and body weight were monitored weekly as previously reported^15^. Fecal samples were collected from mice in the EUG‐H group to validate the role of EUG‐derived bacteria.

In vivo inhibition of the cAMP‐PKA‐CREB pathway. High‐fat diet‐induced NAFLD mice were randomized into three groups (n = 5 per group): HFD, HFD+EUG, and HFD+EUG+H89. EUG was administered at 0.5uL g^−1^ body weight per mice once a day for 3 days. H89 (5 mg kg^−1^) was intraperitoneally injected 30 min prior to EUG administration. Liver tissues were collected 8 h after the last administration of EUG for TG, TC, and gene expression analyses.

‌Fatty acid oxidation assay in primary hepatocytes‌: NAFLD mice received HFD or HFD + EUG (n = 3 per group) for one week. Primary hepatocytes were then isolated. Briefly, mice were rapidly anesthetized, and the liver was perfused via the portal vein first with calcium/magnesium‐free HBSS containing 0.5 mM EDTA and 10 mM HEPES (pH 7.4) (perfusion buffer I) for 2–3 min, followed by perfusion buffer II containing 0.8 mg mL^−1^ collagenase type IV for 10 min. Hepatocytes were released, washed twice, and assessed for viability. FAO assay was subsequently measured in these freshly isolated hepatocytes.

To silence the olfr544 gene, an AAV8 vector expressing a short hairpin RNA (shRNA) targeting mouse Olfr544 (shRNA: ACCTCGGACTCAGCAACAGGTCTTCATCAAGAGTGAAGACCTGTTGCTGAGTCCTTTTT) was constructed. This vector was driven by the TBG promoter to achieve hepatocyte‐restricted expression. Subsequently, each 6‐weeks‐old mice received a single tail vein injection of either AAV‐shOlfr544 or a scrambled shRNA control (AAV‐shScr) at a dose of 1 × 10^11^ viral particles suspended in 100 µL of sterile PBS. Following viral delivery, all mice were subjected to continuous 8 weeks HFD feeding.

GF C57BL/6J mice (7 weeks old) obtained from Kunshan Mingqian Microbiological Research Institute Co., Ltd (Kunshan, Jiangsu, China) were employed to evaluate the gut microbiota‐mediated contribution to the anti‐obesity effects of EUG. GF mice were housed in sterile isolators under controlled conditions. GF status was confirmed monthly through fecal bacterial cultures (aerobic/anaerobic) and 16S rRNA PCR analysis. The animal husbandry and EUG administration procedures followed the established protocols (n = 6/group). The mice were euthanized by cervical dislocation, and liver tissue and blood were immediately snap‐frozen in liquid nitrogen and stored at −80 °C for subsequent analysis.

The mice were euthanized by cervical dislocation for blood collection. Relevant organs were collected and immediately immersed in liquid nitrogen and stored at −80 °C for further analysis. For histological analysis, liver slices were fixed with 4% paraformaldehyde (Servicebio, #G1101). Fresh excreta from animals were harvested and stored at −80 °C for further analysis.

The metabolic energy expenditure parameter was determined using an indirect calorimetry system (Comprehensive Lab Animal Monitoring System, Columbus, America). Briefly, mice were acclimated in training cages for 2 days prior to measurements and were allowed 2 additional days to acclimate to the cabinets. Gas exchanges were recorded every 20 min over 30 h. The results were normalized by weight, and graphs and statistical analysis were obtained using CLAX (v.2.2.16).

### Serum Biomarkers

AST, ALT, TC, TG, HDL, LDL, IL‐6, TNF‐α, IL‐1β in mice serum were determined using an enzyme‐linked immunosorbent assay kit, which was purchased from Nanjing Jiancheng Bioengineering Institute (Nanjing, China).

### Hepatic Biomarkers

Hepatic lipids were extracted from 10% liver tissue homogenates with tert‐butyl alcohol/methanol/Triton X‐100 (3:1:1 v/v/v). Hepatic lipid peroxidation was assessed by quantifying GSH, SOD, and MDA levels. 10% liver homogenate was mixed with 0.1 mL chloroform and then vortexed and centrifuged. Hepatic TG, TC, GSH, MDA, and SOD contents were quantified using assay kits (Jiancheng Bioengineering Institute, Nanjing, China) following the manufacturer's protocols.

### Morphology and Histology

Liver and adipose tissue specimens were fixed in 4% para‐formaldehyde (PFA) for 24 h and then embedded in paraffin. The 5 µm sections were stained with hematoxylin and eosin (H&E) for assessment of histopathological changes. For Oil Red O staining, the fixed liver samples were embedded in optimum cutting temperature compound. Sections of 10 µm were stained with 0.5% Oil Red O solution to assess hepatic lipid accumulation. Whole‐tissue slide scans at 40 × magnification were performed under a microscope slide scanner (Pannoramic MIDI, 3DHISTECH). Relative 20 × and 80 × images were analyzed with image analyzing software CaseViewer_2.3. The staining of Olfr544 (anti‐Olfr544, CABTBL2734, Creative Diagnostics) in liver tissue for immunohistochemical (IHC) analysis was performed following a standard protocol.

### Compositional Analysis of SAVO

The sample solution was obtained by dissolving 20 uL of SAVO in 1.98 mL of ethyl acetate solution and filtering through a 0.22 um filter membrane. The volatile chemical components of SAVO were analyzed using GC‐MS (Agilent 7890A/7000B). The chromatographic conditions were: column Restek Rtx‐5MS (30 m × 250 µm, 0.25 µm); inlet temperature 270 °C; helium carrier gas, and a column flow rate of 1.3 mL min^‒1^. The heating conditions were an initial temperature of 70 °C, held for 3 min at 6 °C min^‒1^ liter to 160 °C, increased to 190 °C at 3 °C min^‒1^, and finally, increased to 230 °C at 10 °C min^‒1^. The mass spectrometry conditional tuning method used was standard spectrogram tuning. The electron ionization mode, electron energy of 70 eV, and an ion source temperature of 230 °C were used. The interface temperature was 280 °C. The spatial distribution of EUG in the liver sections at various time points was analyzed using AFADESI‐MSI, which enables high‐sensitivity and spatially resolved metabolite detection.^[^ ^70^ ^]^ For serum EUG quantification, mice exposed to EUG by inhalation were euthanized by cervical dislocation at designated time points. Serum samples were collected and subjected to GC‐MS analysis. Positive controls (serum‐free matrix spiked with EUG) were included to validate assay specificity and minimize matrix interference.

### Lipids Content and Oxidative Stress Detection

The Hepa1c1c‐7 cell line (RRID:CVCL_0328) was originally sourced from ATCC, procured through ATCC‐authorized distributor Guangzhou Jennio Biotech Co., Ltd. (Lot No.  JNO‐M0145; Cell line were tested and confirmed negative for mycoplasma contamination), and was authenticated by STR profiling with confirmation of no contamination. Cells were cultured in Dulbecco's modified Eagle's medium (Servicebio, China) containing 15% fetal bovine serum (LONSERA, Uruguay) and and 1% penicilline‐streptomycin (Servicebio, China) at 37 °C in incubator containing 5% CO_2_. Lipids content and oxidative stress detection were performed following a standard protocol in Supporting Information.

### Lipase Activity Assay

Lipase activity was determined using a commercial colorimetric assay (Abcam, ab102524), quantifying ATGL‐mediated triglyceride (TAG) hydrolysis by the enzymatic detection of glycerol release. Briefly, Hepa1c1c‐7 cells (10^6^) were suspended in 200 µL of cold 1 × phosphate‐buffered saline and then centrifuged for 5 min at room temperature at 14000 × g to pellet any debris. The clear supernatant was subsequently assayed.

### Fatty Acid Oxidation Activity Detection

Fatty acid oxidation (FAO) activity in Hepa1c1c‐7 cells was assessed using the FAOBlue fluorescent assay. Following 36 h of treatment with FFA in the presence of EUG or EUG plus the adenylyl cyclase inhibitor SQ22536, cells were washed twice with HEPES‐buffered saline (HBS) and incubated with FAOBlue reagent in HBS (37 °C, 50 min). FAO activity was quantified using fluorescence microscopy (excitation/emission wavelength: 405/435 nm).

### shRNA‐Mediated Olfr544 Knockdown

To knockdown the expression of Olfr544 in Hepa1c1c‐7 cells, shRNA (ACCTCGGACTCAGCAACAGGTCTTCATCAAGAGTGAAGACCTGTTGCTGAGTCCTTTTT) was inserted into the shRNA cloning site of the pLKO.1‐EGFP‐puro vector. 293T cells were co‐transfected with psPAX2 and pMD2.G vectors to generate lentivirus‐expressing shRNAs and the recombinant vector described above. After 48 h of transfection, supernatants containing lentivirus were harvested and filtered through 0.45 µm filters. Hepa1c1c‐7 cells were infected with lentivirus for 48 h and selected using puromycin (2.5 µg mL^−1^).

### Protein Extraction, Nucleus and Cytoplasm Separation, and Western Blotting

Proteins from mice liver tissues and Hepa1c1c‐7 cells were extracted with CytoBuster reagent (Sigma–Aldrich) and quantified by a protein assay kit (Bio‐Rad) according to the manufacturer's protocols. Western blotting was performed with the following antibodies: GAPDH (abcam, catalogue Abcam Cat# ab8245, RRID:AB_2 107 448; dilution 1:5000), Olfr544 (Creative Diagnostics catalogue, no. CABT‐BL2734; dilution 1:1000), PKA (abcam, catalogue Abcam Cat# ab75991, RRID:AB_1 524 202; dilution 1:1000), CREB (Cell Signaling Technology, catalogue no. #9197, RRID:AB_331 277; dilution 1:1000), pCREB (Cell Signaling Technology, catalogue no. #9198, RRID:AB_2 561 044; dilution 1:1000). The protein samples were separated by SDS‐PAGE, transferred onto PVDF membranes, and the membranes were blocked in a protein‐free rapid blocking buffer for 4 h. They were then incubated with the primary antibody overnight at 4 °C and HRP‐conjugated anti‐rabbit IgG for 45 min at room temperature. Immunoreactive bands were visualized with an enhanced chemiluminescence (ECL) substrate kit (SQ203L; Epizyme) and analyzed using Image‐Pro Plus 6.0. The protein expression levels were normalized to that of GAPDH.

### Gene Expression Analysis and Quantitative Reverse Transcription‐Polymerase Chain Reaction(RT‐PCR)

The total RNA from liver tissues and hepatocytes were extracted with Trizol reagent (Invitrogen) according to the manufacturer's instructions as previously described.^[^ ^71^ ^]^ Total RNA (1 µg) was used as a template for the reaction. The reverse transcription reaction was carried out with reverse transcription enzyme (Yeasen, Shanghai, China). RT‐qPCR analysis was performed using the SYBR Green PCR master mix (Yeasen) using the ABI7500 real‐time PCR system (Applied Biosystems). The mRNA expression was calculated using the 2^‒ΔΔ^CT quantification method and normalized to that of the housekeeping gene. (All primer messages are shown in Table S2, Supporting Information). Results were presented as a percentage expression of each gene relative GAPDH.

### Cell Transfection

Hana3A cells (obtained as a gift from Prof. Yiqun Yu (Fudan University), originally sourced from Dr. Hiroaki Matsunami's lab (Duke University), RRID: CVCL_RW32; The Cell line has been tested and confirmed negative for mycoplasma contamination.) were seeded into 96‐well plates and cultured overnight in Minimal Essential Medium (MEM) supplemented with 10% fetal bovine serum under standard conditions (37 °C, 5% CO_2_). The transfection of all constructs was performed using Lipofectamine 2000 (ThermoFisher). For each 96‐well plate, a total of 12 µg of receptor plasmid DNA, 2.4 µg of CRE‐Luc, 2.4 µg of mouse RTP1S, and 2.4 µg of pRL‐SV40 were introduced into the cells. Twenty‐four hours post‐transfection, luciferase activity was measured to assess transcriptional activation. Full‐length *Olfr734*, *Olfr43*, *Olfr16*, and *Olfr544* genes were amplified from the mouse liver cDNA library and subcloned into a PCI vector. For alanine‐substitution mutagenesis, site‐directed mutagenesis was performed on the Olfr544 construct to replace specific residues with alanine codons, followed by subcloning into the PCI plasmid.

### Luciferase Assay

The Dual‐Luciferase Reporter Assay System (Cat# RG027, Beyotime) was utilized to measure OR activation via the Gαolf‐mediated AC‐cAMP‐PKA signaling cascade, which phosphorylates CREB and induces firefly luciferase expression under the control of cAMP‐responsive elements. Luminescence signals were quantified using a microplate reader. Cells were co‐transfected with firefly luciferase (cAMP reporter) and Renilla luciferase (constitutively expressed from the SV40 promoter in pRL‐SV40; Promega) to normalize for transfection efficiency and cell viability. Twenty‐four hours post‐transfection, the culture medium was replaced with 100 µL of different concentrations of EUG solutions (diluted in Opti‐MEM, ThermoFisher), followed by a 24‐h incubation (37 °C, 5% CO_2_). Firefly and Renilla luciferase activities were examined with a dual‐luciferase reporter assay. Normalized OR activity was calculated as (LN – Lmin)/(Lmax – Lmin), where LN represents odorant‐induced luminescence, and Lmin and Lmax denote plate background and maximal signals, respectively. Three‐parameter dose‐response curves were fitted using GraphPad Prism 9.0.

### RNA‐seq Data Analysis

RNA‐seq data were analyzed as described previously.^[^ ^71^ ^]^ The date was first trimmed to remove low‐quality bases using trim galore v0.4.4. DESeq2 v1.24.0 was used to perform the differential expression analysis. Gene Ontology enrichment analysis was performed using R package cluster Profiler v3.12.097.

### DNA Isolation, and 16S rRNA Sequencing

Bacteria DNA was extracted from the fecal samples using a QIAamp‐DNA mini stool kit (QIAGEN, CA, USA) according to the manufacturer's instructions. DNA was quantified using agarose gel electrophoresis, and spectrophotometric measurement using a NanoDrop One system (Thermo Scientific, Waltham, USA), respectively, and then stored at −80 °C until further use. For the 16S rRNA amplicon sequencing, the modified paired region‐specific primers (341F‐805R), ≈2–5 µL of extracted DNA, and sequencing primers attached to Illumina paired‐end adapters were used to amplify the V3 and V4 regions of the bacterial 16S rRNA gene using Q5 Hot Start Polymerase (NEB, Ipswich, MA). The abundance and diversity of intestinal flora in mice were determined using Illumina HiSeq sequencing (I‐Sanger, Beijing, China). The QIIME software package was used to conduct the bioinformatic analyses of the sequences as described previously.^[^ ^71^ ^]^ Sequences with a 97% sequence identity were attributed to the same operational taxonomic units (OTUs), OTU table was picked using the open reference picking strategy based on the Greengenes database. QIME was used for the Principal Coordinates Analysis depending on the unweighted unifrac distances, and inter‐group comparisons were conducted using Wilcoxon rank‐sum tests.

### Isolation and Culture of Bacteria and the Single Bacterial Transplantation

2–3 fresh faecal pellets were resuspended by vortexing in anaerobic PBS, and then the supernatant was harvested and coated on MRS medium (Hope Bio‐Technology Co., Ltd., Qingdao, China) to isolate *L. johnsonii* and *L. reuteri*. Strains were identified by 16S sequencing. Finally, *L. johnsonii XR25* and *L. reuteri XR23* were isolated and cultured. They were stored in the Guangdong Microbial Culture Collection Center, and the strain preservation numbers was 65 011 and 65 010, respectively. The bacteria were inoculated in aseptic MRS liquid medium and prepared the bacterial liquid at a concentration of 1 × 10^^9^ cfu mL^−1^ with sterile water. Administer 200 µL of *L. johnsonii* or *L. reuteri* suspension to the respective groups of mice via gastric perfusion once a day for 4 weeks. Additionally, the HFD group mice was provided with an equal amount of normal saline, which served as a control.

*L. johnsonii XR25* and *L. reuteri XR23* were inoculated in MRS liquid medium for 24 h, and then the cultures were centrifuged and supernatants were collected, filtered with a 0.22 µm‐sized pore diameter filter under aseptic conditions. Hepa1c1c‐7 cells were seeded into 6‐well flat‐bottomed plates and modeled as described above, and co‐cultured with 10% cultures’ supernatant for 24 h. The relative indicators were detected using enzyme‐linked immunosorbent assay, oil red staining, and RT‐PCR technology.

### Metabolomics Profiling for Fecal Samples

Metabolomics profiling and the metabolites were identified by searching the self‐built database MWDB (Metware Biotechnology Co., Ltd. Wuhan, China) and public databases (MassBank, KNApSAcK, HMDB, MoTo DB, and METLIN). The details are displayed in Supporting Information.

### Molecular Docking

The crystal structure of Olfr544 (receptor molecule) used for docking was downloaded from the AlphaFold DB model of E9PX47_MOUSE (gene: Or55b4, organism: mouse). The 3D structure of EUG (ligand molecule) was constructed from Chem3D, and energy minimization was carried out under the MMFF94 force field. Blind docking was performed using the CB‐DOCK2 online server (https://cadd.labshare.cn/cb‐dock2/php/blinddock.php). CB‐DOCK2 employs artificial neural network‐based cavity detection and Autodock Vina for molecular docking. The docked complex results were selected based on binding energy, followed by interaction analysis and docking pose evaluation to guide subsequent dynamics simulations and validate binding stability. Ligand‐receptor interactions were analyzed using the PLIP online server (https://plip‐tool.biotec.tu‐dresden.de/plip‐web), and 3D conformations of ligand‐receptor complexes were visualized using PyMOL software.

### Molecule Dynamics

MD simulations were conducted using GROMACS 2023.3 for a total duration of 100 ns, the Generalized Amber Force Field was employed to describe small organic molecules, while the AMBER14SB force field was utilized for protein modeling. Prior to production runs, energy minimization was performed using the mdrun command with the steepest descent algorithm under the canonical (NVT) ensemble, using an initial step size of 0.01 nm and a maximum force tolerance of 1,000 kJ mol^−1^·nm. Following energy minimization, a 100 ps NVT equilibration was implemented at constant volume, gradually increasing the system temperature from 0 to 310.15 K to ensure homogeneous solvent distribution. Subsequently, a 100 ps isothermal‐isobaric ensemble equilibration was conducted using the Berendsen barostat to stabilize the pressure at 1 bar. During the MD production runs, hydrogen bonds were constrained using the LINCS algorithm with a 2‐fs integration time step. Electrostatic interactions were calculated using the Particle‐Mesh Ewald method with a 1.2 nm cutoff. Non‐bonded interactions were truncated at 10 Å and updated every 10 steps. Post‐simulation trajectory analysis included periodicity removal followed by RMSD, RMSF, Rg, and hydrogen bond count evaluations. Detailed protocols for MM/GBSA binding free energy calculations are provided in the Supporting Information.

### Surface Plasmon Resonance

The separation method for membrane fragments and the SPR refers to the established methodology.^[^ ^24^ ^]^ Briefly, membrane fragmentation was performed using the MEM‐PER Plus Membrane Protein Extraction Kit (Thermo Fisher Scientific) according to the manufacturer's instructions. Hana3A cells expressing Olfr544 were harvested by scraping and centrifuged at 300 × g for 5 min. The resulting pellet was washed with Cell Wash Solution, resuspended in Permeabilization Buffer, and incubated at 4 °C for 15 min. Following centrifugation, solubilization was performed, and the supernatant containing solubilized membrane components was collected after additional centrifugation. Membrane fragments isolated from untransfected Hana3A cells served as the negative control.

SPR experiments were conducted using a Biacore T200 system. A carboxymethyl dextran sensor chip (L1; XanTec bioanalytics, Germany) was initially rinsed with HBS‐EP buffer. Subsequent surface modification involved sequential treatment with: a 1:1 mixture of 0.1 M NHS and 0.4 M EDC, 5 mM carbohydrazide, and finally ethanolamine. Membrane fragments were then immobilized onto the L1 chip – specifically, fragments from Olfr544‐expressing cells on flow cell 2 (test group) and fragments from untransfected cells on flow cell 1 (blank control). Immobilized membrane fragments were stabilized with 0.1 mM sodium cyanoborohydride injection. EUG was tested at concentrations of 0.31, 0.62, 1.25, 2.5, 5.0, 10.0, and 20.0 µM using a constant flow rate of 30 µL min^−1^. Bound chemical quantification used ΔRU values, calculated by subtracting reference RU from sample solution RU.

### Statistical Analysis

All statistical analysis was run using the Graphpad Prism 9.0 software (Graphpad). The number of animals and replicate in vivo experiments was specified in each figure legend. *P* values less than 0.05 were considered statistically significant differences as determined by unpaired two‐tailed Students’*t* test, one‐way analysis of variance (ANOVA) or rank sum test. Correlation analyses were performed based on Spearman's statistic performed using the OmicStudio tools at https://www. omicstudio. cn. Statistical significance was set at. ^*^ *p* < 0.05; ^**^ *p* < 0.01; ^***^ *p* < 0.001.

## Conflict of Interest

The authors declare no conflict of interest.

## Author Contributions

X.‐R.W. and Z.‐Z.L contributed equally to the work. Z.‐Z.L., M.‐S.M., and W.‐X.Z. conceived and supervised the research. Z.‐Z.L. and X.‐R.W. analyzed the data, collated the results and wrote the manuscript. W.‐X.Z., X.‐R.W. and Y.‐L.H. were responsible for clinical sample and patient information collection. Z.‐Z.L., M.‐S.M., and X.‐R.W. revised the manuscript. J.‐X.M., X.‐L.Z., W.‐J.C., Q.‐h.W., L.‐y.L. and X.‐R.W. performed the animal experiments assisted by Z.‐Z.L., Y.‐G.S., X.‐X.W. and S.‐D.S. Z.‐Z.L, X.‐R.W., S.‐D.S., Y.‐Z., and Y.‐F. performed all the other experiments, data analysis and prepared the figures and tables. All authors have reviewed and approved the final manuscript.

## Supporting information



Supporting Information

## Data Availability

The data sets used and analyzed during the currentstudy are available from the corresponding author onreasonable request. Data from high‐throughput sequencing were deposited in the NCBI database (SRA Bioproject No. PRJNA1163769, https://www.ncbi.nlm.nih.gov/bioproject/PRJNA1163769/). Supplementary materials (figures, tables, scripts, graphical abstract, slides, videos, Chinese translated version, and update materials) can be found online.
