# Supplementary material for: Inhaling Eugenol Inhibits NAFLD by Activating the Hepatic Ectopic Olfactory Receptor Olfr544 and Modulating the Gut Microbiota
Source: Adv Sci (Weinh). 2025 Aug 20;12(42):e10321. doi: 10.1002/advs.202510321 (PMC12622515; doi:10.1002/advs.202510321)
Supplement: Supplementary file 1 — Supporting Information [file ADVS-12-e10321-s001.docx]

Supplementary Materials for

**Inhaling eugenol inhibits NAFLD by activating the hepatic ectopic olfactory receptor Olfr544 and modulating the gut microbiota**

Xiao-Ran Wang^a,b,1^, Zhan-Zhan Li^c,d,1,*^, Shu-Ding Sun^c,d^, Ya-Gang Song^c,d^, Jin-Xin Miao^c,d^, Xiang-Xiang Wu^c,d^, Yong-Li Han^e^, Xiao-Lei Zhang^c,d^, Wen-Jing Chen^c^, Qing-Hua Wang^f^, Yu-Zhang^c^, Yiping-Fu^c^, Yu-ting Liu^f^, Lin-yan Lang^f^, Wen-Xia Zhao^b*^, Ming-San Miao^c,d*^

^a^*First Clinical Medical College, Henan University of Chinese Medicine, Zhengzhou 450046, China*

^b^*Department of Digestive Diseases, The First Affiliated Hospital of Henan University of Chinese Medicine, Zhengzhou 450046, China*

^c^*Academy of Chinese Medicine Sciences, Henan University of Chinese Medicine, Zhengzhou 450046, China*

^d^*Henan Collaborative Innovation Center for Research and Development on the Whole Industry Chain of Yu-Yao, Zhengzhou 450046, China*

^e^*Acupuncture Department, The First Affiliated Hospital of Henan University of Chinese Medicine, Zhengzhou 450046, China*

^f^*Pharmacy College, Henan University of Chinese Medicine, Zhengzhou 450046, China*

1 Contributed equally.

* Correspondence author: Ming-San Miao: miaomingsan@hactcm.edu.cn; Zhan-Zhan Li: [wind_lz@hactcm.edu.cn](mailto:wind_lz@hactcm.edu.cn); Wen-xia Zhao: [zhao-wenxia@163.com](mailto:zhao-wenxia@163.com)

**Supplementary Methods**

*Lipids content and oxidative stress detection in cell models*

To mimic in vivo hepatic steatosis, Hepa1c1c-7 cells were maintained in medium containing 1.0 mmol/L free fatty acids (FFA, oleic acid and palmitic acid in a ratio of 2:1) for 24h. Hepa1c1c-7 cells were seeded into 6-well flat-bottomed plates at a density of 10,000 cells per well and homogenized by ultrasonication on ice after 24h treatment of different components (50μM), which include the compounds represented by those peaks in Supplementary Table 2 (red peaks). The contents TG, TC, GSH and SOD were determined using the commercial kits (Nanjin Jiancheng Bioengineering Institute, Nanjing, China) according to the manufacturer’s instruction. Meanwhile, intracellular lipid droplet accumulation was assessed by Oil Red O staining (G1262, Solarbio, China) .

*MM/GBSA binding free energy calculations*

The binding free energy between the protein and small molecule was calculated using the MM/GBSA method. The computational formula is expressed as follows:

| ${\Delta G}_{bind}={\Delta G}_{\mathrm{complex}} - ({\Delta G}_{\mathrm{receptor}}+ {\Delta G}_{\mathrm{ligand}})$ |
| --- |
| $={\Delta E}_{\mathrm{internal}}+{\Delta E}_{\mathrm{VDW}}+{\Delta E}_{\mathrm{elec}}{+\Delta G}_{\mathrm{GB}}+{\Delta G}_{\mathrm{SA}}$ |

In this equation, ${\Delta E}_{\mathrm{internal}}$ represents the internal energy, ${\Delta E}_{\mathrm{VDW}}$ represents the van der Waals interaction, and ${\Delta E}_{\mathrm{elec}}$ represents the electrostatic interaction. The internal energies include E_bond_ energy, E_angle_ energy and E_torsion_ energy. ${\Delta G}_{\mathrm{GB}}$ and ${\Delta G}_{\mathrm{GA}}$ are collectively referred to as the solvation free energy. Where ${\Delta G}_{\mathrm{GB}}$ is the polar solvation free energy and Δ*G_SA_* is the non-polar solvation free energy. For ${\Delta G}_{\mathrm{GB}}$, the GB model developed by Nguyen et al[1]. was used for calculation (igb = 2). The non-polar solvation free energy (Δ*G_SA_*) is calculated based on the product of the surface tension (γ) and the solvent accessibility surface area (SA), Δ*G_SA_*= 0.0072 × ΔSASA[2]. Entropy change is ignored in this study due to high computational resource consumption and low accuracy.

*Metabolomics profiling for Fecal Samples*

The fecal sample stored at -80 °C refrigerator was thawed on ice and vortexed for 10 seconds. A 150 μL extract solution (ACN : Methanol = 1:4, V/V) containing internal standard was added into 50 μL sample. Then the sample was vortex for 3 min and centrifuged at 12000 rpm for 10 min (4 °C). A 150 μL aliquots of the supernatant was colleted and placed in -20 °C for 30 min, and then centrifuged at 12000 rpm for 3 min (4 °C). 120 μL aliquots of supernatant were transferred for LC-MS analysis using a ESI-triple quadrupole-linear ion trap (Q TRAP)-MS.

Linear ion hydrazine-flight time (LIT) and triple quadrupole (QQQ) scans were acquired on an API 6500 Q TRAP LC/MS/MS System, equipped with an ESI Turbo Ion-Spray interface, operating in a positive ion mode and controlled by Analyst 1.6 software (AB Sciex, Shanghai, China). The ESI source operation parameters were as follows: ion source, turbo spray; source temperature, 500 °C; ion spray voltage (IS), 5500 V; ion source gas I (GSI), gas II (GSII), curtain gas (CUR) were set at 55, 60, and 25 psi, respectively; the collision gas (CAD) was high. Instrument tuning and mass calibration were performed with 10 and 100 μmol/L polypropylene glycol solutions in QQQ and LIT modes, respectively. QQQ scans were acquired as MRM experiments with collision gas (nitrogen) set to 5 psi. DP and CE for individual MRM transitions were done with further DP and CE optimization. A specific set of MRM transitions were monitored for each period according to the metabolites eluted within this period.

For the MWDB, it was constructed based on the standard materials and purified compounds. Additionally, some publicdatabases also contain some information of metabolites that can be referenced directly. The metabolites were identified by comparing the accurate precursor ion (Q1) andproduction (Q3) values, retention time, and fragmentation pattern with the database. Substance identification has three levels (1) : Level 1, where the sample substance's secondary mass spectrum and RT match with a database substance score of 0.7 orabove; Level 2, where the sample substance's secondary mass spectrum and RT match with a database substance score between 0.5 and 0.7; Level 3, where the sample substance's Q1 (precursor ion), Q3 (product ion), RT, de-clustering potential (DP), collision energy (CE) match consistently with a database substance. Quality control (QC) was generate by pooling equal aliquot of each sample and was processed together with actual sample. QC was injected every ten samples in the analytical sequence to check the robustness of the nontarget metabolomics workflow.

Unsupervised PCA (principal component analysis) was performed by statistics function prcomp within R (www.r-project.org). The data was unit variance scaled before unsupervised PCA.

For two-group analysis, differential metabolites were determined by VIP (VIP > 1) and P-value (P-value < 0.05, Student’s t test). VIP values were extracted from OPLS-DA result, which also contain score plots and permutation plots, was generated using R package MetaboAnalystR. The data was log transform (log) and mean centering before OPLS-DA. In order to avoid overfitting, a permutation test (200 permutations) was performed.

*Detection of 5-HIAA and IPA in clinical* serum *samples*

Serum samples were prepared and extracted. 5-HIAA and IPA were measured using the ESI-triple quadrupole-linear ion trap (Q TRAP)-MS as above. Quantifications were performed by using external standard method (ESTD).

**Supplementary Table 1. Clinical and biochemical characteristies of the patients with biopsy proven MAFLD**

| **Characteristics** | **All** | **NAFLD** | **Control** | ***P* value** |
| --- | --- | --- | --- | --- |
|  | (n=55) | (n=27) | (n=28) |  |
| **Demographics** |  |  |  |  |
| Age (years) | 48.98±12.33 | 49.11±11.24 | 48.86±13.71 | 0.9403 |
| Gender, n (%) |  |  |  |  |
| Female | 29 (47.37) | 10 (42.86) | 19 (52.94) |  |
| Male | 26 (52.63) | 18 (57.14) | 8 (47.06) |  |
| **Biological data** |  |  |  |  |
| AST (U/L) | 23.78±7.56 | 29.29±7.91 | 19.18±3.41 | ＜0.0001 |
| ALT (U/L) | 25.34±12.41 | 35.69±8.95 | 15.37±5.16 | ＜0.0001 |
| Triglycerides (mmol/L) | 1.53±0.68 | 1.99±0.63 | 1.10±0.39 | ＜0.0001 |
| Total cholesterol (mmol/L) | 4.89±1.13 | 5.33±1.37 | 4.47±0.63 | 0.0052 |
| Glucose (mmol/L) | 5.18±0.96 | 5.37±1.25 | 5.00±0.54 | 0.1755 |
| HDL(mmol/L) | 1.23±0.21 | 1.16±0.21 | 1.31±0.20 | 0.0092 |
| LDL(mmol/L) | 3.12±0.91 | 3.49±1.13 | 2.79±0.47 | 0.0053 |

**Supplementary Table 2.** The primers used in qPCR analysis

| Target | Primer | Sequence (5’-3’) |
| --- | --- | --- |
| GAPDH | Forward | CCTCGTCCCGTAGACAAAATG |
|  | Reverse | TGAGGTCAATGAAGGGGTCGT |
| Olfr544 | Forward | ATCCTCTTCACCAATGCCCTG |
|  | Reverse | ACATCTGTCCCAAGCATCGAG |
| Olfr43 | Forward | TGGGAGTTACAAGGCAGCG |
|  | Reverse | TGGAGGCGAGTGTCAGAGT |
| Olfr16 | Forward | TCACAGCAATGGGTTATGATCG |
|  | Reverse | AGTGCCTAAAGACCCACAAACC |
| Olfr734 | Forward | ATTCCTCTATCACAGCGCCTAAG |
|  | Reverse | CCGACAAAGTGCAAGAAGAAGAG |
| Olfr767 | Forward | GCACCTTCACACTCCCATGT |
|  | Reverse | AGGACATAGCAGCCAGGAGA |
| Olfr107 | Forward | CTGATGTCCACCTCCACACC |
|  | Reverse | ATCTCCAGTCCACAGAGCCT |
| PPARα | Forward | TTTCACAAGTGCCTGTCTGTCG |
|  | Reverse | TCTTCAGGTAGGCTTCGTGGAT |
| CPT-1 | Forward | GCCTCTATGTGGTGTCCAAGTATC |
|  | Reverse | CACCATAGCCGTCATCAGCAA |
| CYP7A1 | Forward | GCTAAGGAGGACTTCACTCTACACC |
|  | Reverse | TGGTCTTTGCTTTCCCACTTTC |
| Cox7A1 | Forward | TTCCAGGCCGACAATGACC |
|  | Reverse | CCCAGAGTCAGCGTCATGG |
| PPARγ | Forward | AGGGCTCGGAACTCCAGAAA |
|  | Reverse | CCAGGGAATCGGTAGACATCG |
| FAS | Forward | CTGCCTCTGGTGCTTGCT |
|  | Reverse | ACCCGCCTCCTCAGCTTT |
| SCD-1 | Forward | GTTAGCACCTTCTTGCGATACACT |
|  | Reverse | GTGAAGTTGATGTGCCAGCG |
| Cidea | Forward | TGCACAGATGACGGGACAG |
|  | Reverse | GTGCTAGGCTTGGGGGATG |
| 16S RNA | 27F | CGGTGAATACGTTCYCGG |
|  | 1492R | GGWTACCTTGTTACGACTT |
| GGI-58 | Forward | GGTTAAGTCTAGTGCAGCGTTTG |
|  | Reverse | GTACTCTGTCACCGTGTCATCTT |
| ATGL | Forward | CCAACATTATTGAGGTGTCCAAGG |
|  | Reverse | AGTGGGATATGATGACGTTCTCTC |
| HSL | Forward | GGAAGGACAGGACAGCAAGGTA |
|  | Reverse | GAGGTAGGGCTCGTGGGATTTA |
| AHR | Forward | CTACTCCACTTCAGCCACCCTC |
|  | Reverse | GCAAGAAGCCGGAAAACTGTC |
| TSC2 | Forward | AGTGGATGGATGTTGGCTTGT |
|  | Reverse | CAGGGTGATAATGAACAGAGGC |
| UCP-1 | Forward | CAAGCGTACCAAGCTGTGCG |
|  | Reverse | CATGATGACGTTCCAGGACCC |

**Supplementary Table 3.** GC-MS analysis of volatile chemical components in SAVO

| Peak | Time | Nmae | Molecular formula | CAS |
| --- | --- | --- | --- | --- |
| 1 | 3.093 | (1R)-2,6,6-Trimethylbicyclo[3.1.1]hept-2-ene | C10H16 | 7785-70-8 |
| 2 | 3.287 | Camphene | C10H16 | 79-92-5 |
| 3 | 4.595 | α-Phellandrene | C10H16 | 99-83-2 |
| 4 | 4.899 | Caryophyllene oxide | C15H24O | 1139-30-6 |
| 5 | 5.01 | (+)-4-Carene | C10H16 | 29050-33-7 |
| 6 | 5.127 | o-Cymene | C10H14 | 527-84-4 |
| 7 | 5.299 | Eucalyptol | C10H18O | 470-82-6 |
| 8 | 5.42 | D-Limonene | C10H16 | 5989-27-5 |
| 9 | 6.23 | γ-Terpinene | C10H16 | 99-85-4 |
| 10 | 6.972 | Cyclohexene, 1-methyl-4-(1-methylethylidene)- | C10H16 | 586-62-9 |
| 11 | 10.447 | Anethole | C10H12O | 104-46-1 |
| 12 | 11.705 | Eugenol | C10H12O2 | 97-53-0 |
| 13 | 12.597 | Caryophyllene | C15H24 | 87-44-5 |
| 14 | 12.985 | Humulene | C15H24 | 6753-98-6 |
| 15 | 13.661 | Phenol, 2-methoxy-4-(2-propenyl)-, acetate | C12H14O3 | 93-28-7 |
| 16 | 14.653 | 2-Cyclohexen-1-ol, 1-methyl-4-(1-methylethyl)-, cis- | C10H18O | 29803-82-5 |

**Supplementary figures**


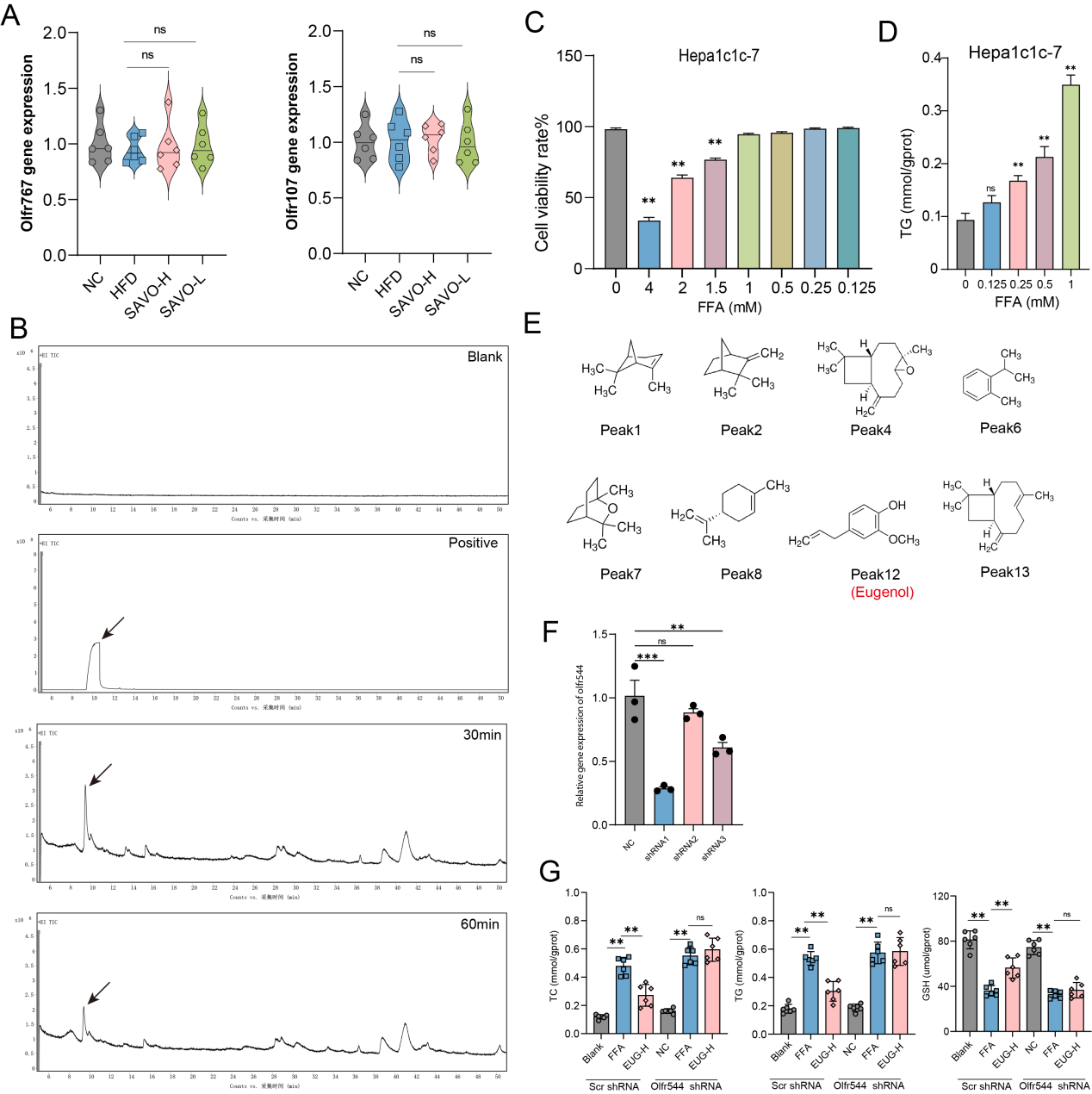


**Fig. S1** **FFA-induced lipid accumulation models in Hepa1c1c-7 cells was established and the knockdown of hepaitc olfr544.**

1. Olfactory receptors mRNA expression in liver (n=6). (B) GC-MS was employed to determine the EUG content in mouse blood at different time points following inhalation. (C) Cell viability rate in different concentrations of FFA. The y-axis represents survival rate. (D) Intracellular TG levels in different concentrations of FFA.
2. Chemical Structures of the first eight components in SAVO. (F) Relative gene expression of Olfr544 in the Hepa1c1c-7 cells from different groups. (G) Intracellular TG、TC、and GSH contents in Hepa1c1c-7 cells with Olfr544 gene knockdown. (H) Lipid synthesis, lipolysis and oxidation relative genes expression in the livers. Data are presented as the means ± SEMs . **p* < 0.05, ***p* < 0.01,****p* < 0.001, ns *p*＞ 0.05.


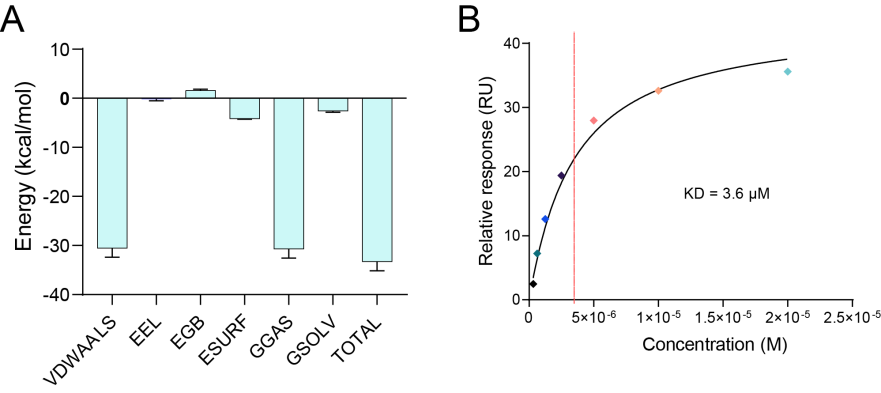


**Fig. S2** **Olfr544 is a receptor of EUG.**

(A) MM/GBSA binding free energy calculations over the entire simulation. (B) Four-parameter curve in SPR analysis.


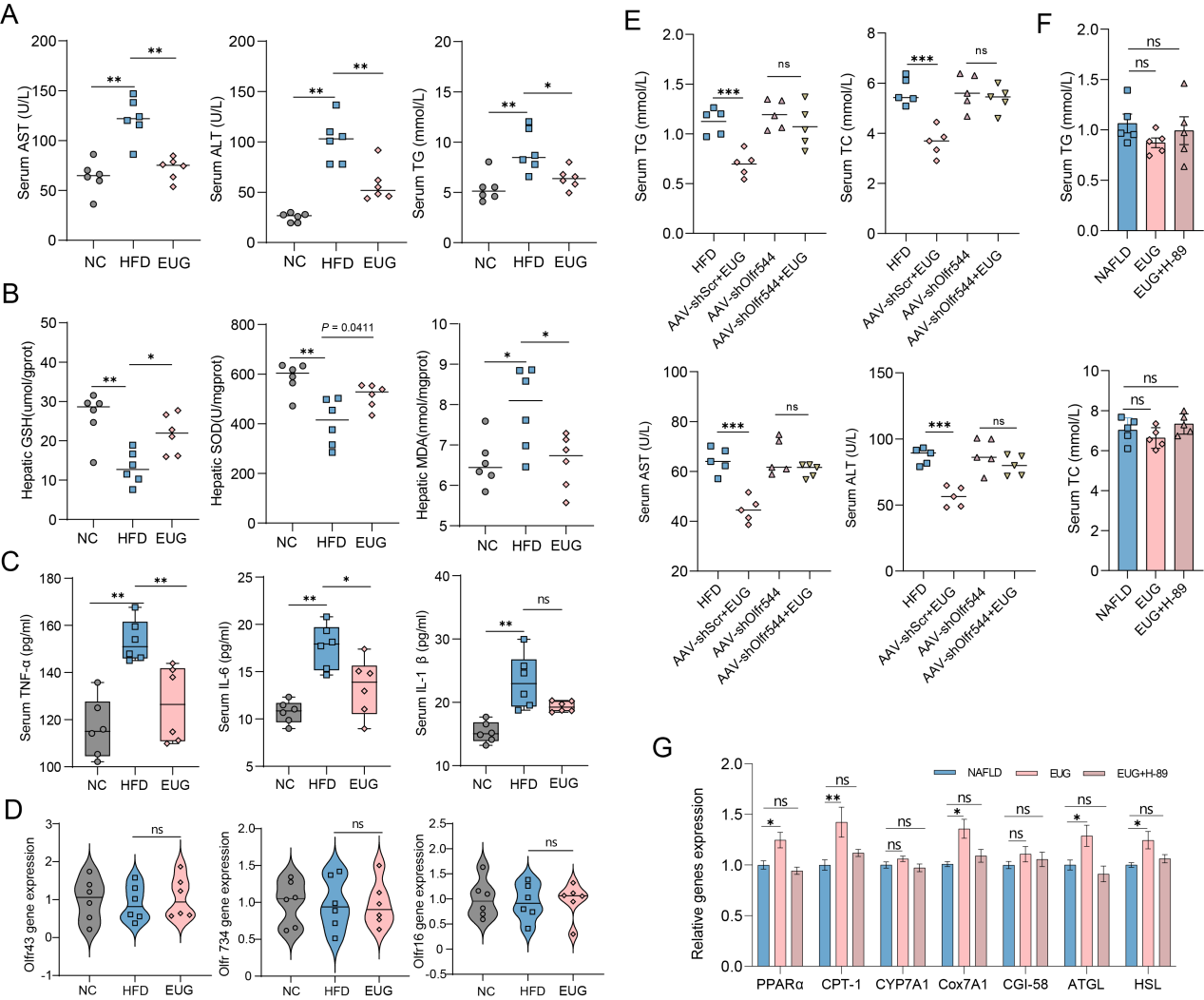


**Fig. S3** **Inhaling EUG suppresses the evolution of NAFLD in HFD-fed mice**.

(A) Serum AST, ALT, and TG levels (n = 6/group) in wild-type mice. (B) Hepatic GSH, SOD, and MDA contents (n = 6/group) in wild-type mice. (C) Serum inflammatory factors were analyzed by ELISA (n = 6/group) in wild-type mice. (D) Olfactory receptors mRNA expression in the liver (n = 6/group). (E) Serum TC, TG, AST, and ALT levels (n = 5/group) in genetically engineered mice. (F) Serum TC, and TG levels (n = 5/group). (G) Lipid lipolysis and oxidation relative genes expression in the livers. Data are presented as the means ± SEMs . **p* < 0.05, ***p* < 0.01,****p* < 0.001, ns *p*＞ 0.05.


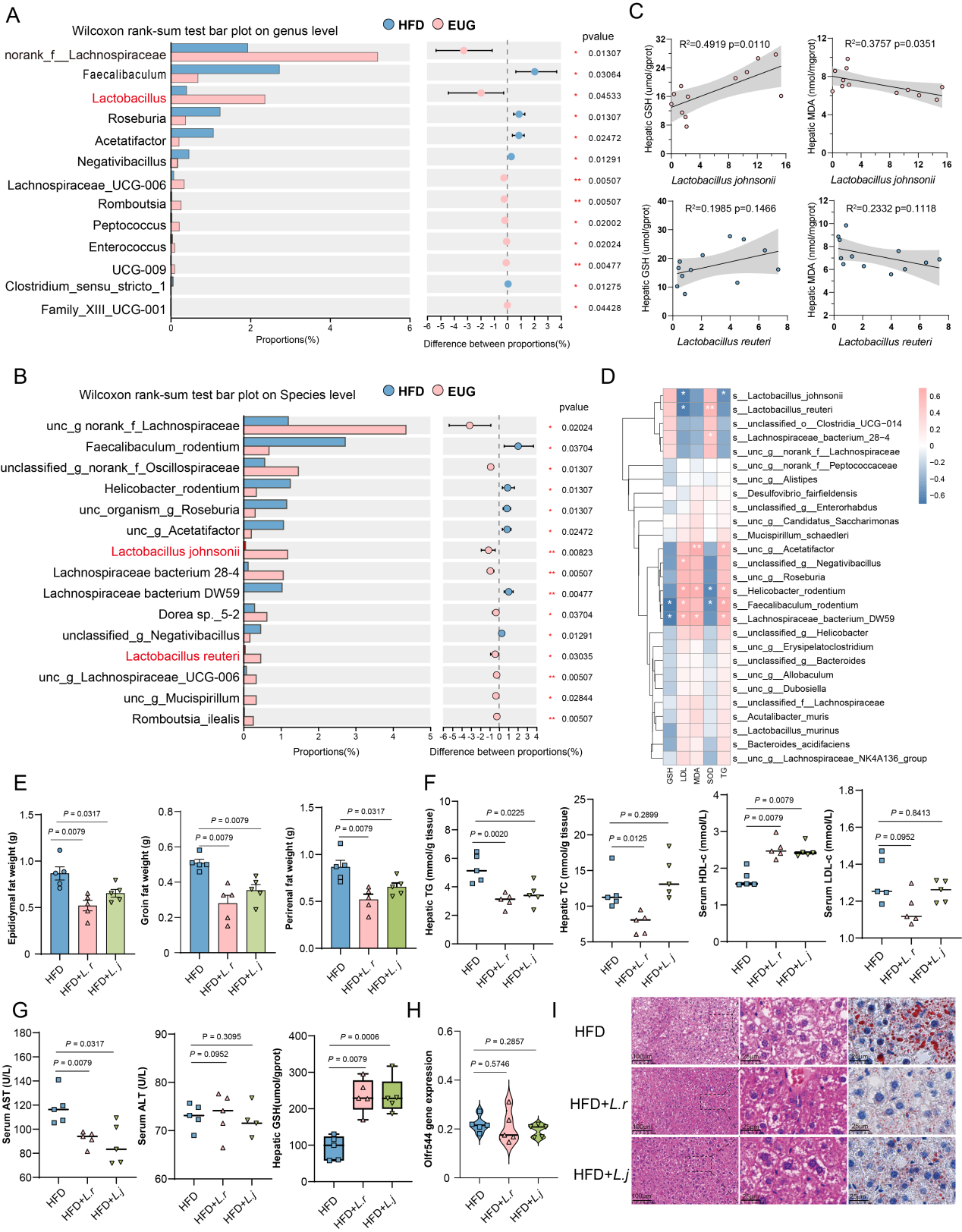


**Fig. S4** **Gut microbiota modulated by GPEO suppress the lipogenesis of NAFLD mice.**

(A, B) Wilcoxon rank-sum test bar plot on genus and species level in HFD and GPEO-H treated mice. (C) Correlation analysis of hepatic GSH and SOD levels with the abundance of L. reuteri and L. johnsonii in bacterias treated mice. (D) Correlation analysis between clinical factors and metabolites by partial Spearman’s correlation. (E) Epididymal, inguinal and perirenal fat weight weigh in HFD and bacterias treated mice. (F) Hepatic TG and TC, Serum HDL-c and LDL-c levels in HFD and bacterias treated mice. (G) Serum TG, TC, and hepatic GSH contents in HFD and bacterias treated mice. (H) Olfr544 gene expression in the livers of HFD and bacterias treated mice. (I) Representative images of H&E and Oil-red O staining of liver sections (original magnification 100 and 200). E-H, n=6; Data are presented as the means ± SEMs . **p* < 0.05, ***p* < 0.01.


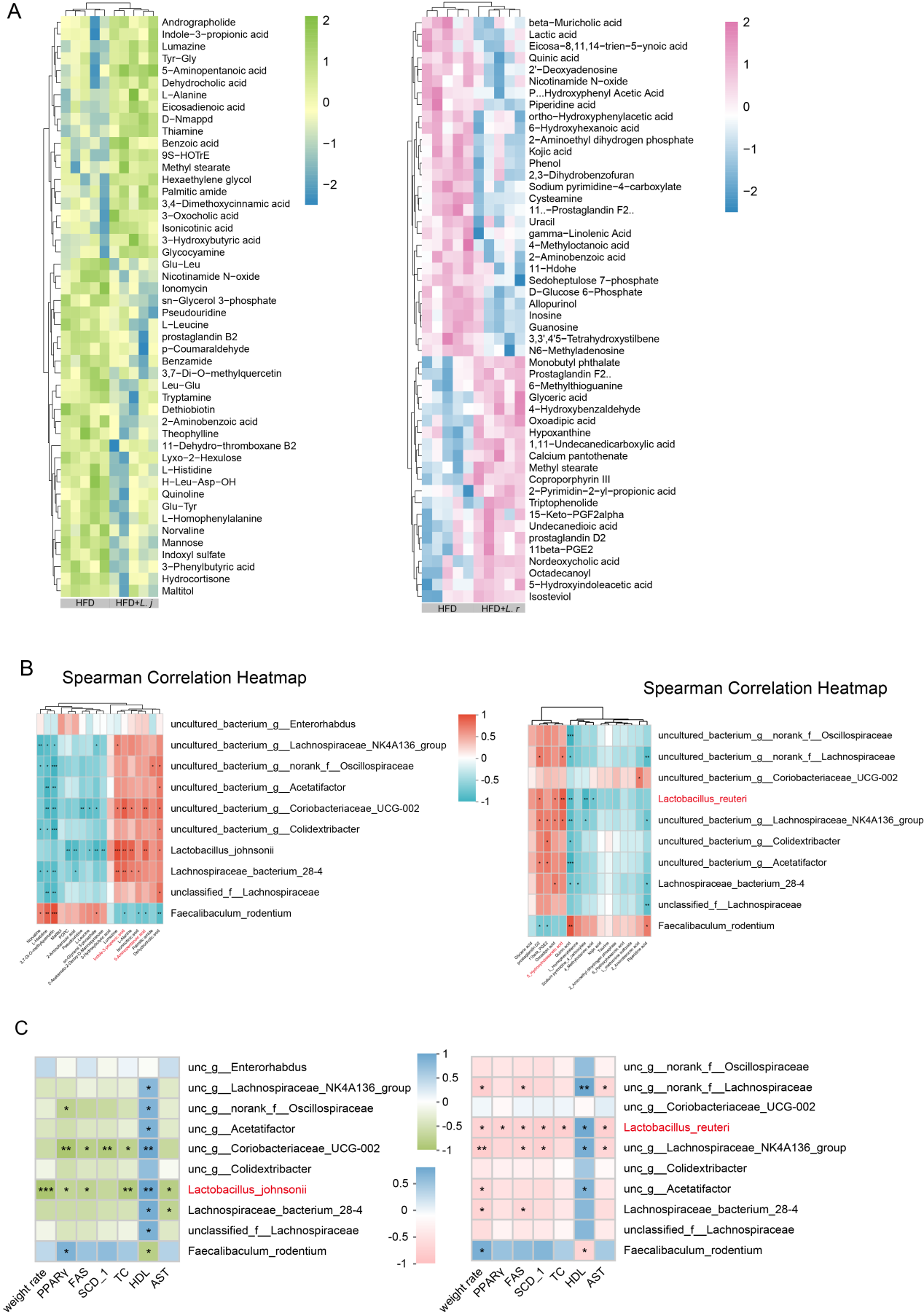


**Fig. S5 Mining of bacterial metabolites.**

1. Heatmap of representative faecal metabolites in mice treated by *L. reuteri* XR23 and *L. johnsonii XR25*. (B) Correlation analysis between the altered TOP 10 bacteria in gut of mice after single bacteria transplantation and the metabolite, which were shared between the metabolites significant altered in mouse fecal contents after bacterial treatment and the cultural supernatan. (C) Correlation analysis between clinical factors and the altered TOP 10 bacteria in gut of mice after single bacteria transplantation.


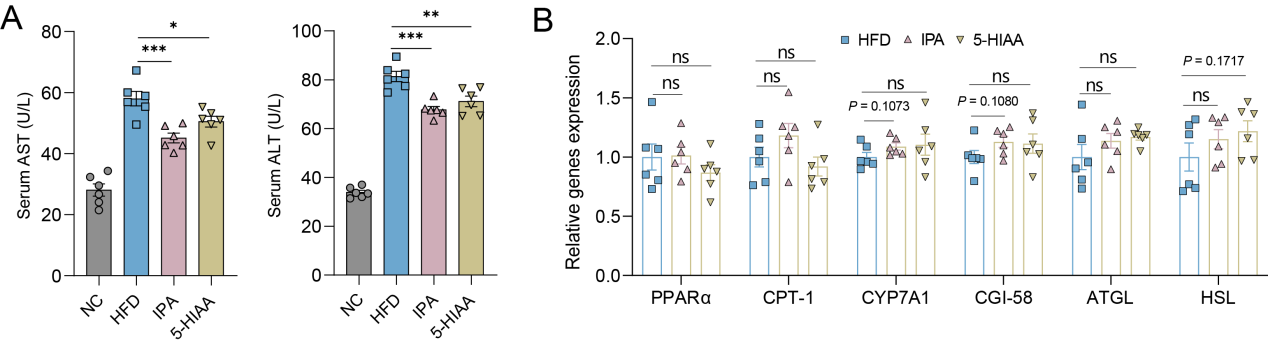


**Fig. S6 The microbial metabolites IPA and 5-HIAA can ameliorate NAFLD.**

(A) Serum AST, and ALT levels (n = 6/group) in mice. (B) Lipid lipolysis and oxidation relative genes expression in the livers. Data are presented as the means ± SEMs . **p* < 0.05, ***p* < 0.01,****p* < 0.001, ns *p*＞ 0.05.

Reference

[1] Rastelli G, Del Rio A, Degliesposti G and Sgobba M. Fast and accurate predictions of binding free energies using MM-PBSA and MM-GBSA. J Comput Chem 2010;31(4):797-810.

[2] Nguyen H, Roe DR and Simmerling C. Improved Generalized Born Solvent Model Parameters for Protein Simulations. J Chem Theory Comput 2013;9(4):2020-34.
